# Repeated exposure to either consistently spatiotemporally congruent or consistently incongruent audiovisual stimuli modulates the audiovisual common-cause prior

**DOI:** 10.1038/s41598-022-19041-7

**Published:** 2022-09-15

**Authors:** Fangfang Hong, Stephanie Badde, Michael S. Landy

**Affiliations:** 1 Psychology Department, New York University, New York, NY USA; 2 Psychology Department, Tufts University, Medford, MA USA; 3 Center for Neural Science, New York University, New York, NY USA

**Keywords:** Psychology, Human behaviour, Neuroscience, Auditory system, Visual system

## Abstract

To estimate an environmental property such as object location from multiple sensory signals, the brain must infer their causal relationship. Only information originating from the same source should be integrated. This inference relies on the characteristics of the measurements, the information the sensory modalities provide on a given trial, as well as on a cross-modal common-cause prior: accumulated knowledge about the probability that cross-modal measurements originate from the same source. We examined the plasticity of this cross-modal common-cause prior. In a learning phase, participants were exposed to a series of audiovisual stimuli that were either consistently spatiotemporally congruent or consistently incongruent; participants’ audiovisual spatial integration was measured before and after this exposure. We fitted several Bayesian causal-inference models to the data; the models differed in the plasticity of the common-source prior. Model comparison revealed that, for the majority of the participants, the common-cause prior changed during the learning phase. Our findings reveal that short periods of exposure to audiovisual stimuli with a consistent causal relationship can modify the common-cause prior. In accordance with previous studies, both exposure conditions could either strengthen or weaken the common-cause prior at the participant level. Simulations imply that the direction of the prior-update might be mediated by the degree of sensory noise, the variability of the measurements of the same signal across trials, during the learning phase.

## Introduction

Imagine walking your dog. It suddenly escapes the leash to chase a squirrel. While anxiously searching for your dog, you see movement behind a bush and also hear a shuffling sound coming from adjacent bushes. Should you aim for a point in between the two bushes or sprint towards one of them? The brain has to make countless decisions like this in daily life as it is common for sensory measurements to be in conflict due to internal noise in the brain and external noise in the environment. To form a coherent percept by integrating the measurements, the brain bases the final location estimate of the dog on a weighted mixture of both visual and auditory measurements^1–3^. Given that compared to audition, vision usually provides less variable, less uncertain spatial information^4^, the brain assigns a higher weight to the visual measurement to locate the dog. This phenomenon—vision dominating the combined audiovisual spatial estimate of the sound source—is called the ventriloquism effect^5,6^.

To continue with the dog-searching example, if the movements came from a bush distant from the shuffling noise, then it would be more sensible to sprint towards one bush, that is, to not integrate these two measurements as they likely have been produced by two different causes. Numerous studies have found evidence for a breakdown of integration when cross-modal measurements are sufficiently discrepant^7–16^. These findings suggest that when making a perceptual judgment, the brain infers the causal relationship underlying the cross-modal measurements. Körding and colleagues^17^ formalized how an ideal Bayesian observer makes a perceptual judgment using causal inference. This Bayesian causal-inference model captures human behavior in a variety of multisensory tasks^8,17–28^. According to this model, the observer derives the posterior probability of the two measurements coming from a common source. This probability is proportional to the product of the likelihood of the common-cause scenario given the sensory measurements and a common-cause prior. This prior represents accumulated knowledge about the probability that cross-modal measurements come from the same source, and it exerts a top-down influence on perception. A strong common-cause prior leads to integration of cross-modal measurements even in the presence of large conflicts between them; a weak common-cause prior leads to segregation of measurements even when the measurements are largely in agreement.

Bayesian priors fall into two broad categories, structural and contextual^29^. Structural priors typically are thought of as reflecting natural statistics and acquired through life-long implicit learning^30–37^. For instance, we perceive a view of the inside of a Halloween mask as convex, rather than having the veridical concave interpretation, due to an overriding prior that faces are convex^38^. Structural priors are assumed to be general in nature and remain stable. In contrast, people also have priors that are context-specific and can be modulated rather rapidly^21,39–41^. This plasticity of contextual priors is supported by evidence that humans are able to learn priors based on novel statistics^30,42–47^ and update existing priors after repeated exposure to new stimulus statistics^23,29,48–51^. The goal of this study was to determine whether the common-cause prior for audiovisual stimuli adapts to new audio-visual stimulus statistics.

Two previous studies have examined the plasticity of the common-cause prior in the context of stimulus statistics^52,53^. Both studies found that a brief exposure to new stimulus statistics can lead to a modulation of the common-cause prior. However, the results of these two studies are not consistent and seem to contradict each other. We re-examined this question with an improved experimental design that accounts for individual spatial biases across vision and audition and participant-level analyses using Bayesian models. First, we ensured that the audiovisual stimuli were perceptually aligned by measuring and adjusting the stimuli based on each participant’s audiovisual spatial bias. Second, we measured participants’ audiovisual spatial perception before and after exposure to new stimulus statistics. Participants localized one modality of an audiovisual stimulus, followed by a unity judgment indicating whether they perceived the stimuli as originating from one or two separate sources. Third, we exposed participants to spatially and temporally congruent audiovisual stimuli in one session and spatially and temporally incongruent audiovisual stimuli in another session. Fourth, we compared the fit of several Bayesian causal-inference models that differed in the degree to which the models assumed a stable common-cause prior. Finally, we ran simulations to scrutinize the update of the common-cause prior during the exposure period and revealed that the direction of the update depends on the degree of variability of sensory measurements of the same object across multiple presentation of the object.

**Figure 1:**
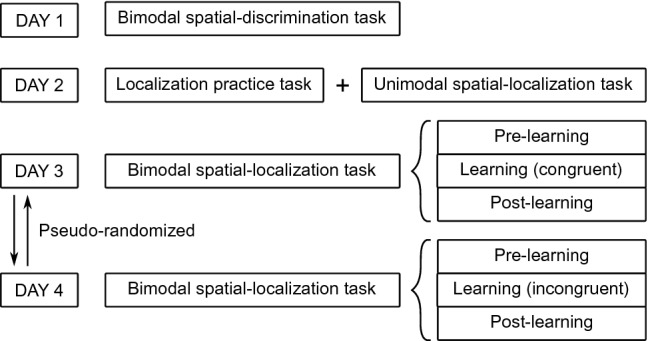
Task order across days.

## Results

In the learning phase of the main experiment, participants were exposed to audiovisual stimulus pairs with artificially manipulated stimulus statistics. Specifically, the audiovisual stimulus pairs were either consistently congruent (temporally and spatially aligned in perceptual space) or incongruent (temporally and spatially misaligned). To examine whether the exposure to new stimulus statistics modulated participants’ common-cause prior, we measured their spatial perception of audiovisual stimulus pairs with variable spatial discrepancies before and after the exposure and fitted variations of the Bayesian causal-inference model to these data. Prior to the main experiment, participants completed three preparatory experiments (Fig. 1) that allowed us to select audiovisual stimuli adjusted for perceptual biases for the main experiment as well as constraining the model parameters.

**Figure 2:**
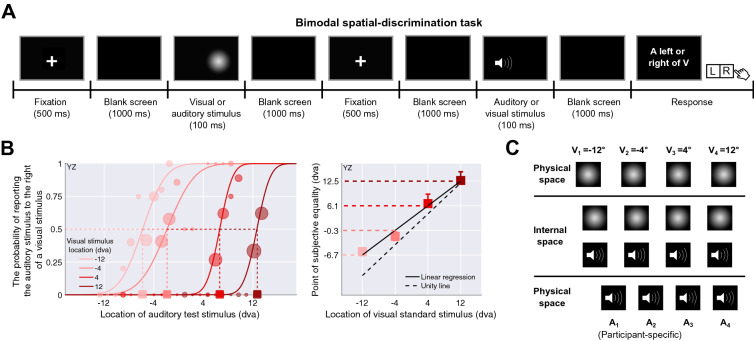
Experimental procedure and behavioral results: bimodal spatial-discrimination task, preparatory experiment 1. (**A**) Task timing. Participants were presented with a visual standard and an auditory test stimulus in random order. After stimulus presentation, they reported whether they had perceived the auditory stimulus to the left or right of the visual stimulus. Feedback was not provided. (**B**) Behavioral results of representative participant YZ. Left panel: binned data (dots; bin size $\approx 1^\circ$; marker area proportional to the number of trials in each bin), and best-fitting psychometric functions (curves) for four visual standard stimulus locations (shades of red). Squares: points of subjective equality (PSE). Right panel: PSE as a function of visual stimulus location. Error bars: 95% bootstrapped confidence intervals (some are smaller than the marker size). Black dashed line: identity line; black solid line: linear regression line fitted to the PSEs; red dashed lines: the locations of the four auditory stimuli perceived as aligned with the four visual standard locations according to the regression line. (**C**) Stimulus locations in physical and perceptual space. Top panel: four visual stimulus locations in physical space used in all experiments; middle panel: the four pairs of a visual and an auditory stimulus that were aligned in internal perceptual space; bottom panel: the four participant-specific auditory stimulus locations in physical space.

### Behavioral results

#### Modality-specific spatial biases (preparatory experiment 1)

The data from this experiment were used to estimate participants’ modality-specific localization biases for auditory relative to visual stimuli. Participants performed a bimodal spatial-discrimination task; they judged whether an auditory test stimulus, presented at various locations, was located to the left or right of a visual standard stimulus presented at one of four locations (Fig. 2A). From those binary discrimination responses, we calculated the point of subjective equality (PSE), the auditory stimulus location that participants perceived to be spatially aligned with one of the four visual stimulus locations. To reduce the influence of measurement noise, we extracted the auditory stimulus locations that were perceptually aligned with the four visual locations from a linear regression line through the four PSEs (Fig. 2B; see Appendix S1 for group data). These participant-specific audiovisual location pairs were used in the subsequent tasks to ensure that congruent audiovisual stimuli were indeed perceived as spatially congruent (Fig. 2C).

**Figure 3:**
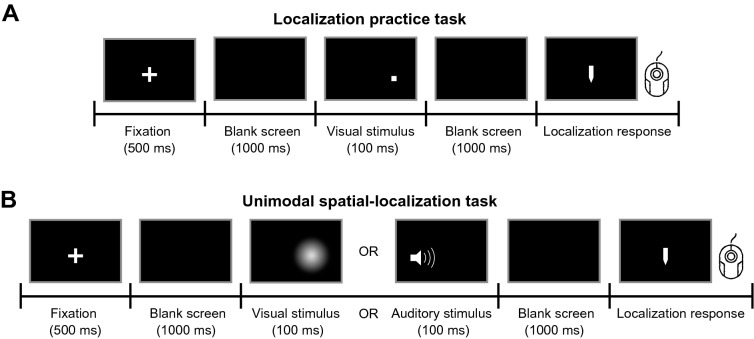
Experimental procedure: localization-practice and unimodal spatial-localization tasks, preparatory experiments 2 and 3. (**A**) Localization-practice task. Participants were presented with a small white square and used a visual cursor to indicate the stimulus’ horizontal location. (**B**) Unimodal spatial-localization task. Participants localized either a unimodal visual or auditory stimulus. Feedback was not provided for either task.

#### Localization-response variability (preparatory experiment 2)

In the next part of the study, participants familiarized themselves with the device used to make spatial-localization responses in all subsequent experiments. We additionally used the data from this task to estimate the variability of participants’ responses independent of the variability introduced by noisy stimulus perception. To this aim, we used visual stimuli for which participants’ localization was highly precise (Fig. 3A). We assumed that localization error was unbiased and localization variability was independent of stimulus location. Thus, we fitted the localization errors with a Gaussian probability density centered at zero. The standard deviation of participants’ (non-outlier) localization responses was $1.59^\circ$ on average (range: 0.83$^\circ$–$2.52^\circ$).

#### Unimodal auditory and visual localization response variability (preparatory experiment 3)

In the next part of the study, participants localized unimodal auditory and unimodal visual stimuli presented at the locations identified in preparatory experiment 1 (Fig. 3B). The direct localization responses in this task were used to validate the modality-specific biases measured in the first preparatory experiment using a spatial discrimination task. For most participants, the localization biases were replicated (Appendix S2). Additionally, we used the mean of these unisensory localization responses for the calculation of the behavioral ventriloquism effects and to constrain several parameter estimates during model fitting.

**Figure 4:**
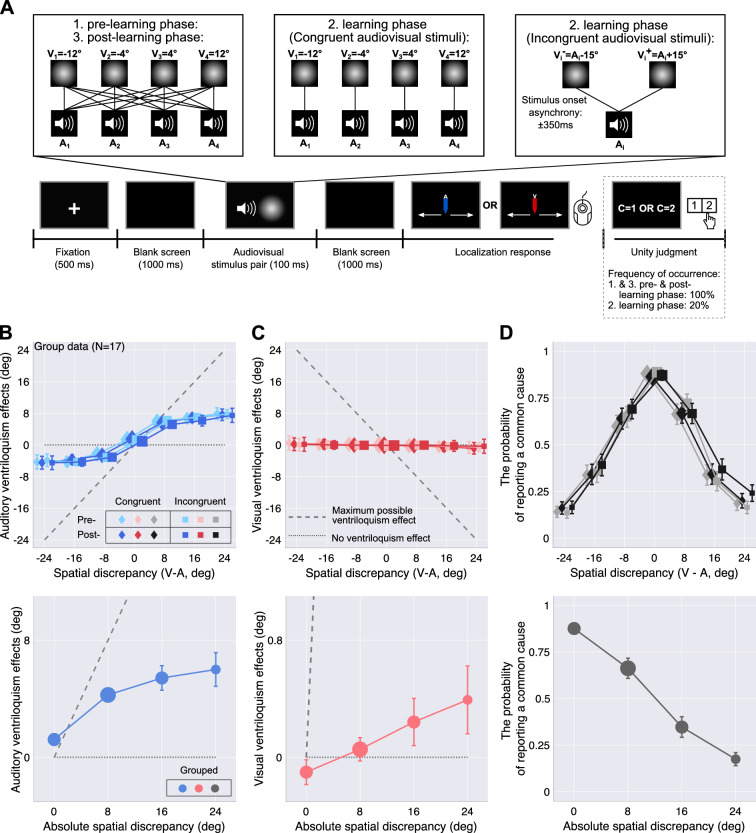
Experimental procedure and behavioral results: bimodal spatial-localization task, main experiment. (**A**) Task timing. Participants were presented with an audiovisual stimulus pair; they indicated the location of one modality specified by a color cue. Subsequently, participants reported whether they perceived the two stimuli to stem from a common source (C $=$ 1) or two separate sources (C $=$ 2) by button press. Sixteen pairs of varying spatial discrepancy were presented during pre- and post-learning phases. Four perceptually matched audiovisual pairs were presented in the congruent learning phase, and 16 spatiotemporally misaligned pairs in the incongruent learning phase. (**B**) Auditory ventriloquism effects, i.e., shifts of auditory localization responses towards the simultaneously presented visual stimulus as a function of spatial discrepancy (top panel) and absolute spatial discrepancy (bottom panel). Data are split by whether the task was conducted before (light hues) or after (darker hues) the learning phase and by the stimulus statistics during the learning phase (marker shape). Error bars: ±SEM. Marker area is proportional to the number of trials. Data are jittered slightly along the x-axis for legibility. (**C**) Visual ventriloquism effects, i.e., shifts of visual localization responses towards the simultaneously presented auditory stimulus. (**D**) The proportion of common-cause reports as a function of spatial discrepancy (top panel) and absolute spatial discrepancy (bottom panel). Note that the y-axis scales are greatly exaggerated in the lower panels of (**B**).

#### Bimodal spatial localization before and after exposure to congruent or incongruent stimulus pairs (main experiment)

Before and after a learning phase, participants localized audiovisual stimuli with varying spatial discrepancies and made an explicit causal inference judgment about the audiovisual stimulus pair by reporting the number of perceived sources (Figs. 1,  4A). These data were used to estimate the common-cause prior during each phase via fitting Bayesian causal-inference models. During the learning phase, participants were exposed to audiovisual stimuli with consistent stimulus statistics with respect to a common cause for audition and vision. Specifically, participants were exposed only to spatially and temporally aligned audiovisual stimulus pairs (congruent condition) in one session, and only to spatially and temporally misaligned audiovisual stimulus pairs in the other session (incongruent condition). Participants completed the congruent and incongruent learning phases in randomized order.

Before fitting models to the data, we performed a classical statistical analysis to verify the existence of ventriloquism effects, shifts in localization responses towards the location of the other modality in audiovisual compared to unisensory trials, and to check for group-coherent modulations of these ventriloquism effects by our experimental manipulations. We used (generalized) linear mixed models to test whether auditory and visual ventriloquism effects as well as the proportion of affirmative unity judgments changed as a function of the absolute spatial discrepancy between auditory and visual locations, the type of learning phase (congruent vs. incongruent), and the phase (pre- vs. post-learning). For the auditory ventriloquism effect, only a significant main effect of absolute spatial discrepancy emerged ($\chi ^2(3) = 891.94$, $p<0.001$; linear contrast: $\beta =3.32$, $t = 12.03$; quadratic contrast: $\beta = -0.92$, $t = -3.68$; cubic contrast: $\beta = 0.25$, $t = 1.12$; Fig. 4B, bottom panel); see Appendix S3.1 for the full statistical model). Post-hoc comparisons indicated a significant difference of the auditory ventriloquism effect from zero at absolute spatial discrepancies of $0^\circ$ ($t(16) = 2.95$, $p = 0.01$, $d = 0.72$), $8^\circ$ ($t(16) = 10.98$, $p < 0.001$, $d = 2.66$), $16^\circ$ ($t(16) = 6.26$, $p < 0.001$, $d = 1.52$), and $24^\circ$ ($t(16) = 5.05$, $p < 0.001$, $d = 1.23$). For the visual ventriloquism effect only significant main effects of absolute spatial discrepancy ($\chi ^2(3) = 102.86$, $p < 0.001$; linear contrast: $\beta = 0.45$, $t = 5.89$; quadratic contrast: $\beta = 0.02$, $t = 0.24$; cubic contrast: $\beta = -0.03$, $t = -0.47$; Fig. 4C, bottom panel), and condition ($\chi ^2(1) = 18.83$, $p < 0.001$) emerged (Appendix S3.2). Visual ventriloquism effects were not significantly different from zero at any absolute spatial discrepancy ($0^\circ$: $t(16) = -1.16$, $p = 0.26$; $8^\circ$: $t(16) = 0.67$, $p = 0.51$; $16^\circ$: $t(16) = 1.45$, $p = 0.17$; $24^\circ$: $t(16) = 1.65$, $p = 0.12$).

For the binary unity judgments, only significant main effects of absolute spatial discrepancy ($\chi ^2(3) = 4428.39$, $p < 0.001$; linear contrast: $\beta = -2.90$, $z = -31.43$, $p < 0.001$; quadratic contrast: $\beta = 0.17$, $z = 2.20$, $p = 0.03$; cubic contrast: $\beta = 0.16$, $z = 2.57$, $p = 0.01$), and condition ($\chi ^2(1) = 14.56$, $p < 0.001$; $\beta =0.16$, $z = 2.53$, $p = 0.01$) emerged (Fig. 4D, bottom panel; Appendix S3.3).

In sum, while we reliably replicated visual-auditory ventriloquism effects, no significant effects of the learning phase emerged. However, it is impossible to determine whether the common-cause prior changed during the learning phase based on the statistical results of the implicit and explicit causal-inference tasks as responses in these tasks depend on the common-cause prior in a non-linear way that is modulated by the stimulus characteristics (for simulations see Ref. 8). Thus, next, we fitted causal-inference models to each participant’s data.

### Modeling results

#### Models involving the common-cause prior

To test whether the common-cause prior was modulated after repeated exposure to consistent stimulus statistics, we fitted several variants of the Bayesian causal-inference model of multisensory integration^17^ to our data. The model variants we tested differed in terms of the plasticity of the common-cause prior (Table 1). The high-plasticity, short-lasting changes model assumes that the common-cause prior is malleable and thus changes after the exposure to consistently congruent/incongruent stimuli. This model additionally assumes that the changes dissipate relatively quickly once participants complete a session and re-experience natural stimulus statistics outside of the laboratory, that is, the common-cause prior will be restored back to the baseline level when participants come in for the second session. Thus, this model assumes three common-cause priors, one for the two pre-learning phases before adapting to new stimulus statistics, and two for the post-learning phases, after adapting to either congruent or incongruent stimulus statistics. We also considered a high-plasticity, long-lasting changes model, which also assumes that the common-cause prior is plastic but the changes either take a relatively long time to dissipate or are context-specific and thus partially recovered once the participant returns to the experiment room for the second session. This model assumes four common-cause priors, one each for the pre-learning and post-learning phases accompanying the congruent and incongruent learning phases. These two high-plasticity models were contrasted against a no-plasticity model, which assumes that the common-cause prior is not affected by short-term changes in the stimulus statistics, and thus the exposure to consistently congruent/incongruent audiovisual stimuli is not sufficient to modulate the prior. This model assumes only one common-cause prior, shared by the pre- and post-learning phase of both sessions (Table 1).

**Table 1:** Common-cause priors in the three tested models.

Phase	Condition	Model		
		High-plasticity, short-lasting changes	High-plasticity, long-lasting changes	No-plasticity
Pre-learning	Congruent	$p_{C=1,\text {pre}}$	$p_{C=1,\text {pre, cong}}$	$p_{C=1}$
	Incongruent		$p_{C=1,\text {pre, incong}}$	
Post-learning	Congruent	$p_{C=1,\text {post, cong}}$	$p_{C=1,\text {post, cong}}$	
	Incongruent	$p_{C=1,\text {post, incong}}$	$p_{C=1,\text {post, incong}}$	
Number of common-cause priors		3	4	1

**Figure 5:**
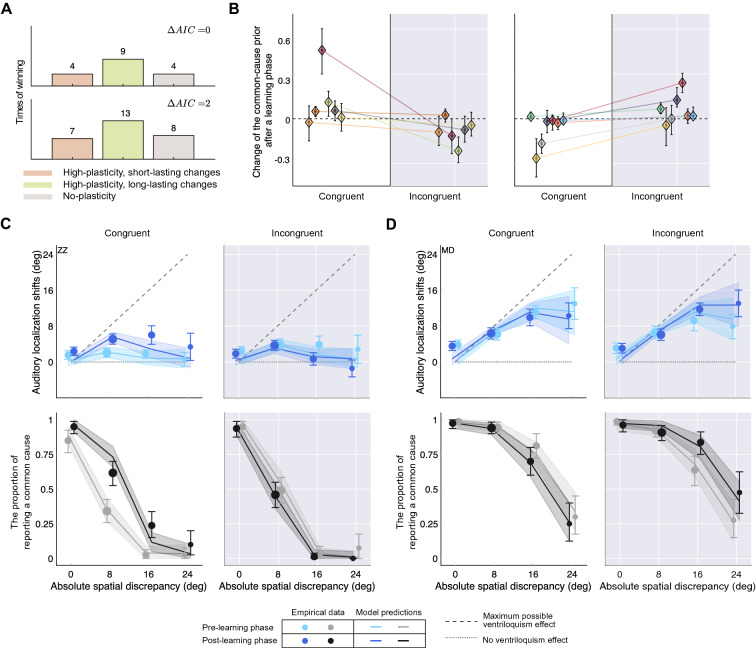
Model comparison and parameter estimates. (**A**) Results of two different model comparisons. Top panel: using a conservative criterion, there is only one best model, the one with the minimal AIC value; bottom panel: a liberal criterion selecting all models with an AIC value not exceeding the minimal AIC value by more than 2. (**B**) Change of the common-cause prior from the pre- to the post-learning phase as estimated by the best-fitting model. Color indicates individual participants. Participants are grouped according to the effect of the exposure phase on their common-cause priors (left panel: adaptation to the stimulus statistics, i.e., increases/decreases of the common-cause prior after congruent/incongruent learning phases, right panel: the opposite pattern to that in the left panel). Error bars: 95% bootstrapped confidence intervals. Dashed line: no change of the common-cause prior after the learning phase. (**C**) Empirical data (dots) and model predictions (lines) for representative participant ZZ from the first group in (**B**). Auditory ventriloquism effects (top panels) and the proportion of common-cause reports (bottom panels) are plotted as a function of absolute spatial discrepancy for the pre- (lighter color) and post-learning phase (darker color). Error bars: ±2 SEM (top panel) and 95% binomial confidence intervals (bottom panels); shaded areas: 95% confidence intervals computed by parametric bootstrapping. (**D**) Empirical data and model predictions for representative participant MD from the second group in (**B**). Please see Appendix S4 for all participants.

#### Model comparison, parameter estimates and model predictions

The two high-plasticity models captured the data of the majority of participants (Fig. 5A). This indicates that the common-cause prior is not a structural prior but plastic. It can be modulated by brief exposure to consistently spatiotemporally congruent or incongruent audiovisual stimulus pairs.

However, the changes of the common-cause prior from the pre-learning to the post-learning phase were inconsistent across participants, whether congruent or incongruent stimuli were presented during the learning phase (Fig. 5B). Some participants (N $=$ 6) adapted the common-cause prior in the direction of the stimulus statistics. They showed a significant increase in the common-cause prior after the congruent learning phase, and/or a decrease in the common-cause prior after the incongruent learning phase (Fig. 5B, left panel; Fig. 5C, see Appendix S4.1 for all participants falling into this category). Yet, many other (N $=$ 7) participants’ common-cause prior changed in the opposite direction: it decreased after the congruent learning phase and/or increased after the incongruent learning phase (Fig. 5B, right panel; Fig. 5D, see Appendix S4.2 for all participants falling into this category). In summary, the common-cause prior can be flexibly modulated after a brief exposure to either spatiotemporally congruent or incongruent audiovisual stimuli, but the direction of the update varies across participants; it either goes in the direction of the stimulus statistics or against it.

**Figure 6:**
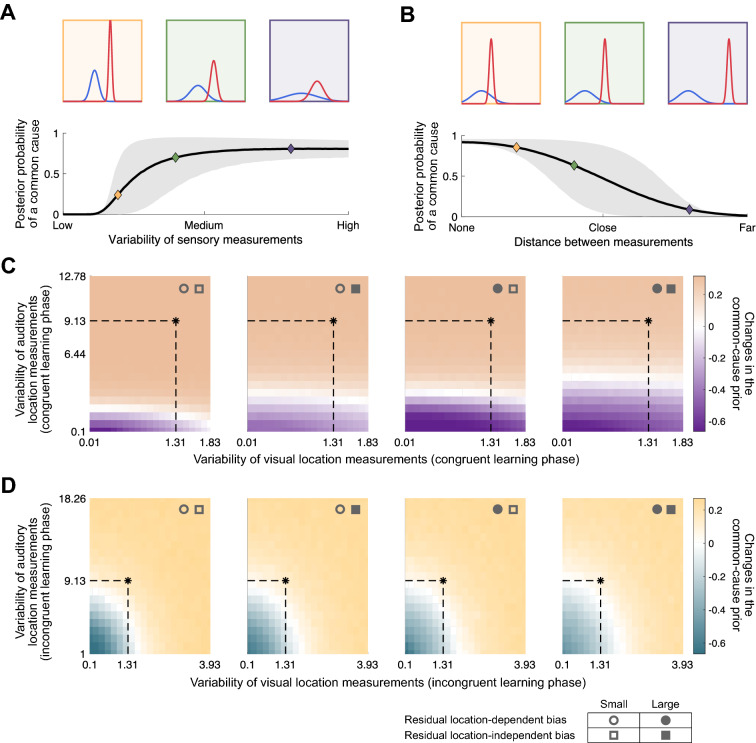
Simulation results. (**A**) Influence of the variability of auditory (blue) and visual (red) location measurements on the posterior probability of a common cause. Shaded area: ±SD. Both uncertainties increase along the horizontal axis. Three pairs of auditory and visual likelihood functions are simulated, ranging from relatively low (orange), to medium (green), and high (purple) variability of both sensory measurements. (**B**) Influence of the distance between auditory and visual location measurements on the posterior probability of a common cause. Three pairs of auditory and visual likelihood functions are simulated, from perfectly aligned measurements (orange), to close proximity (green), and highly discrepant measurements (purple). (**C**) Accumulated updates of the common-cause prior, averaged across 100 simulations of the congruent learning phase, as a function of visual (x-axis) and auditory (y-axis) measurement variability. Each panel shows a different residual misalignment of auditory relative to visual spatial perception. Open/filled circles represent small/large residual location-dependent bias (0.75/0.5 times the mean estimated location-dependent bias); open/filled squares represent small/large residual location-independent bias (the bias is $3^\circ /6^\circ$ more leftward compared to the mean estimated location-independent bias); asterisks and dashed lines represent group means of the estimated measurement variability, which was assumed to be the same across the pre- and post-learning phases. (**D**) Simulated accumulated updates of the common-cause prior after the incongruent learning phase.

### Simulation results

To examine the factors that could potentially mediate the updating of the common-cause prior, we simulated this process over the course of both congruent and incongruent learning phases. For the simulations, we made three assumptions: (1) After each encounter with an audiovisual stimulus pair, the common-cause prior is updated in the direction of the posterior probability that the auditory and visual location measurements came from the same source^8,21,26^. (2) The sensory noise, the variability of auditory and visual measurements across trials, differs across participants and changes with the stimulus statistics. Specifically, it is different between congruent and incongruent learning phases in which extreme audiovisual stimulus statistics were selected; it is also different between learning and pre- and post-learning phases in which audiovisual stimulus pairs of varying spatial discrepancies were presented. We cannot estimate the degree of sensory noise during the learning phases due to sequential dependencies but our simulations allow us to relate it to our estimates of the variability of visual and auditory measurements during pre- and post-learning phases. For the congruent learning phase, sensory noise levels comparable to those estimated for the pre-and post-learning phases would lead to updates of the common-cause prior in the direction of the stimulus statistics. To replicate updates of the common-cause prior contradictory to the stimulus statistics, the variability of the measurements needed to be smaller than in the pre- and post-learning phases (Fig. 6C), which is in agreement with the literature (see “Discussion”). For the incongruent learning phase, the group mean of the estimated measurement variability during the pre- and post-learning phases is right at the border between sensory noise levels associated with updates in the direction of the stimulus statistics and sensory noise levels associated with updates in the opposite direction (Fig. 6D). Thus, the interindividual differences in the direction of the update of the common-cause prior can already be explained by interindividual differences in sensory noise. (3) In addition, we included location-independent (constant) or location-dependent (proportional) offsets between the perceived locations of presumably congruent visual and auditory stimuli in our simulations. Slight offsets were required to simulate changes of the common-cause prior contradicting stimulus statistics in congruent conditions, yet, residual offsets might have been present in our study despite our efforts to identify perceptually aligned stimulus pairs. In sum, stimulus statistics manipulated during the learning phase are not the only driving factor that updates the common-cause prior; our simulations show that the level of sensory noise and biases in audiovisual spatial perception also play critical roles in the posterior probability of a common cause (Fig. 6A,B, for further simulations see Refs. 8, 21), which in turn determines the updates of the common-cause prior.

## Discussion

In this study, we investigated the plasticity of the common-cause prior for vision and audition. To modulate the common-cause prior, we exposed participants to either consistently spatiotemporally congruent or consistently incongruent audiovisual stimuli. To estimate changes in the common-cause prior, we measured participants’ audiovisual spatial perception before and after exposure to the new stimulus statistics and fitted with Bayesian causal-inference models of multisensory integration. The behavior of the majority of the participants was best captured by two model variants that assume a plastic common-cause prior with changes lasting either for short or long periods. This indicates that the common-cause prior is flexible—it can be modulated quickly after a brief exposure to consistent stimulus statistics. Interestingly, the direction of modulation of the common-cause prior varied across participants. For some participants, the common-cause prior was updated in the direction of the stimulus statistics during the learning phase, for others, the updates shifted the prior in the opposing direction. We suggest that this heterogeneity is due to interindividual differences in sensory noise, in the variability of sensory measurements of the same object across repeated presentations.

Modeling the data at the individual level allowed us to scrutinize differences between participants. We observed changes in the common-cause prior indicating adaptation to the new stimulus statistics for some participants. For other participants, however, changes in the common-cause prior went in the opposite direction compared to the new stimulus statistics. Together, these diverse changes observed across participants provide an explanation for contradictory group-level findings in previous studies^52,53^. Odegaard and colleagues^52^ reported that on a group level the common-cause prior was strengthened significantly after brief exposure to synchronous audiovisual stimuli that were spatially incongruent and temporally congruent or correlated. No significant change of the common-cause prior was found in conditions in which the audiovisual stimuli were spatiotemporally congruent. Tong and colleagues^53^ investigated the plasticity of the common-cause prior indirectly by comparing ventriloquism effects measured after exposure to consistently spatiotemporally congruent audiovisual stimuli to ventriloquism effects measured after exposure to consistently spatiotemporally incongruent audiovisual stimuli. They found larger ventriloquism effects after exposure to congruent than after exposure to incongruent audiovisual stimuli, which were interpreted as indicative of changes in the common-cause prior in the direction of the stimulus statistics in one or both of the conditions. Both studies relied on group-level analyses. Our finding indicates large interindividual differences in the updates of the common-cause prior. A group-level analysis may well pool participants with significant changes in the common-cause prior in both directions, and thus the group effects will depend on the vagaries of sampling participants. Moreover, differences in the stimuli between the two experiments might have affected stimulus reliability, which our simulations have shown to be a crucial determinant of updates of the common-cause prior.

Intuitively one would expect the common-cause prior to strengthen after repeated encounters with congruent stimuli and to weaken after frequent encounters with incongruent stimuli; such a behavior would indicate adaptation of the prior to changing stimulus statistics in the environment. For this reason, it is perplexing that we and others^52^ found changes of the common-cause prior in the opposite direction of the new stimulus statistics. Yet, the vast majority of our participants showed a consistent pattern across the two conditions suggesting that this is a systematic effect and not due to noise in the estimation of the common-cause prior (which we verified to be low in a separate study; Hong, Xu, Kalia, Badde, & Landy, VSS abstract). To explore which factors could plausibly lead to the counterintuitive changes of the common-cause prior, we simulated the updates of the prior for each learning condition. The simulation results indicate that one of the major driving factors of the updates of the common-cause prior is the level of sensory noise during the learning phase. Interindividual differences in sensory noise were sufficient to explain updates in different directions during the incongruent learning phase. To replicate counterintuitive updates against the stimulus statistics during the congruent learning phase we needed to assume a reduction in sensory noise during this phase compared to pre-and post-learning phases in which audiovisual stimulus pairs with varying spatial discrepancies were presented. Consistent with this assumption, the variability of sensory measurements has been found to decrease after exposure to congruent stimuli, an effect known as multisensory enhancement^23,54–60^.

In our simulations, decreases in the common-cause prior after exposure to consistently congruent stimuli were additionally bound to residual perceptual misalignments between congruent stimuli. Perceptual misalignments between physically aligned audiovisual stimuli certainly exist and, in line with previous studies^5,21,61,62^, we found peripheral biases in auditory relative to visual spatial perception (Appendix S1). We compensated for these biases by presenting individually determined, perceptually aligned audiovisual stimuli. However, our efforts might not have been perfectly successful; we measured the biases twice, once using an audiovisual discrimination task and once using a unimodal direct localization task and the estimated biases show some inconsistencies across these tasks (Appendix S2). Importantly, in the case of sensory measurements with very little variability, small perceptual offsets might have large consequences in the congruent learning condition as our simulation shows.

In sum, we suggest that interindividual differences in the variability of sensory measurements, which is contingent on stimulus statistics, determine in which direction the common cause-prior is updated. Confirming this hypothesis via modeling requires including the data from the learning phase while model fitting. However, doing so is computationally challenging as the learning phase itself is characterized by sequential dependence of the updates of the common-cause prior and therefore a lack of a closed-form solution for the model. Confirming this hypothesis experimentally would require a design that allows us to keep track of the measurement variability throughout the learning phase. For example, one could require participants to make unimodal and/or bimodal localization judgments interspersed throughout the learning phase, although this would make a long learning session even longer.

### Conclusion

This study examined the plasticity of the common-cause prior for vision and audition. To this aim, we repeatedly presented participants with either spatiotemporally aligned or misaligned audiovisual stimulus pairs, and measured participants’ auditory and visual spatial perception before and after the exposure. We then fitted variants of the Bayesian causal-inference model and found that models assuming different common-cause priors for the pre- and the post-learning phases captured the behavior of the majority of the participants, indicating that the common-cause prior can be dynamically adapted when new audiovisual stimulus statistics are encountered. However, for some participants the common-cause prior changed in a counterintuitive way: it decreased after exposure to congruent stimuli or increased after exposure to incongruent stimuli. Our simulations attribute these various patterns of results to individual levels of sensory noise during the learning phase.

## Methods

### Participants

Seventeen participants (ten females, aged 20–33, mean: 26, all right-handed), recruited from New York University and naive to the purpose of the study, participated in all experiments. All stated they were free of visual, auditory, and motor impairments. The data of three additional participants (one male and two females, aged 25–33, mean: 28, one left-handed) were excluded from data analysis and model fitting due to a conflict between the modality-specific spatial biases indicated by the bimodal spatial-discrimination task and by the unimodal spatial-localization task (Appendix S2). Experimental protocols were approved by the Institutional Review Board at New York University, and all procedures were conducted in accordance with the relevant guidelines and regulations of the Institutional Review Board, and in accordance with the Declaration of Helsinki. All participants gave informed consent prior to the beginning of the study. Sixteen participants were compensated $12 per hour and one participant opted out of compensation.

**Figure 7:**
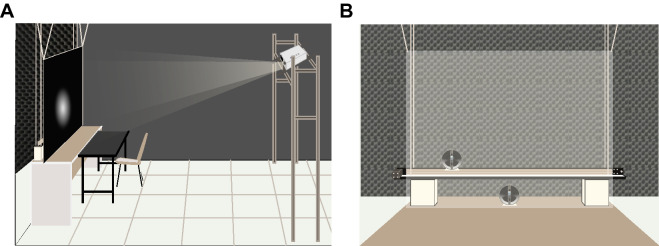
Experimental setup. (**A**) Side view of the experiment room. Participants sat in front of a large white screen, which was hung from the ceiling using elastic ropes. An LCD projector was mounted above and behind participants to project visual stimuli on the screen. (**B**) Front view of the experiment room. Behind the white screen (the transparency was changed for the purpose of visualization), there was a loudspeaker mounted on a sledge attached to a linear rail. The rail was elevated from the table and hung from the ceiling using elastic ropes. A second loudspeaker was located behind the screen straight ahead of the participant.

### Apparatus and stimuli

The experiments were conducted in a dark and semi sound-attenuated room (Fig. 7A). Participants were seated 1 m from an acoustically transparent, white screen ($1.36 \times 1.02$ m, $68 \times 52^\circ$ visual angle) with their chins resting on a chinrest. An LCD projector (Hitachi CP-X3010N, 1024 $\times$ 768 pixels, 60 Hz) was mounted above and behind participants to project visual stimuli onto the screen. The default visual stimulus was a high-contrast (36.1 cd/$\text {m}^2$) Gaussian blob (SD: $3.6^\circ$) presented on a black background. Only in the localization practice task, the second preparatory experiment, the default visual stimulus was replaced with a white square ($8 \times 8$ pixels $\approx 0.6 \times 0.6^\circ$). For experiments that involved spatial localization responses, a response cursor, a rectangle ($6 \times 12$ pixels) on top of an upside-down triangle ($6 \times 12$ pixels), was presented during the response phase.

Behind the screen, a loudspeaker (20 W, ${4}{\Omega }$ full-range speaker, Adafruit, New York) was mounted on a sledge attached to a linear rail (1.5 m long). The rail was hung from the ceiling using elastic ropes; it was located 23 cm above the table and 5 cm behind the screen, perpendicular to the line of sight (Fig. 7B). The position of the sledge on the rail was controlled by a microcomputer (Arduino Mega 2560; Arduino, Somerville, MA, USA). The microcomputer activated a stepper motor that rotated a threaded rod (OpenBuilds, https://www.openbuildspartstore.com), which allowed for horizontal movement of the speaker. The speaker was moved to its predetermined location before an auditory stimulus was presented. Auditory stimuli were 100 ms-long noise bursts (0–20.05 kHz, 60 dB), windowed using the first 100 ms (the positive part) of a sine wave with a period of 200 ms.

We were concerned that participants might infer the position of the speaker from the noise created by the speaker’s movements. This strategy was foiled by playing a masking sound during each movement of the speaker; the masking sound was played through an additional loudspeaker, positioned just behind the center of the screen. The masking sound (55 dB) consisted of a recording of the sound generated by a randomly chosen speaker movement plus white noise. To further foil the use of auditory cues from the moving sledge, every time the speaker moved to a new target location, it first moved to a stopover location. The stopover location was randomly chosen under the constraint that the total distance the speaker moved and the amount of time the movement took were approximately equal across trials. The combination of these two approaches successfully prevented participants from inferring the speaker position based on sounds arising from the movements of the speaker on the rail^21^.

Responses were given via a numeric keypad or a wireless rollerball mouse. Stimulus presentation, speaker movement, and data collection were controlled by an iMac running MATLAB R2017b (MathWorks, Natick, MA, USA). Visual stimuli were presented using the Psychophysics Toolbox^63,64^. All analysis was performed using MATLAB 2020b and R 4.0.2.

### Procedure

#### Preparatory experiment 1: bimodal spatial-discrimination task

In this 2-IFC task, participants judged the location of an auditory stimulus relative to that of a visual standard stimulus. The two stimuli were presented sequentially, in pseudo-randomized order. Each trial started with the presentation of a fixation cross located at the center of the screen for 500 ms, followed by a blank screen lasting for 1000 ms. Then, the first, 100 ms-long stimulus was presented, followed by a blank screen presented for 1000 ms. After the first stimulus presentation, the same events (fixation, blank screen, second stimulus) were repeated. At the end of each trial, a response probe was displayed. Participants indicated by button press whether the auditory stimulus was located to the left or right of the visual stimulus. Feedback was not provided (Fig. 2A). The inter-trial interval was approximately 3000 ms. The long period was needed to move the loudspeaker to its new position for the next trial.

The visual stimulus was presented at one of four locations, $\pm 4$ or $\pm 12^\circ$ relative to the center of the screen. The location of the auditory test stimulus was controlled by eight interleaved staircases, two for each visual stimulus location. Of the two staircases, one started the auditory test stimulus from the left side, and the other from the right side of the visual standard stimulus (for a visual standard stimulus located at $-12^\circ$, the left staircase starts at $13.25^\circ$ and the right staircase starts at $1.25^\circ$; the other triplets are {$V_2 = -4^\circ$, $A_{2,\text {left}}: -9.25^\circ$, $A_{2,\text {right}}: 8.75^\circ$}, {$V_3 = 4^\circ$, $A_{3,\text {left}}: -8.75^\circ$, $A_{3,\text {right}}: 9.25^\circ$}, {$V_4 = 12^\circ$, $A_{4,\text {left}}: -1.25^\circ$, $A_{4,\text {right}}: 13.25^\circ$}). Staircases starting from the right side followed the one-down-two-up rule, converging to a probability of 29% of choosing the auditory stimulus as farther to the right than the corresponding visual standard stimulus. Staircases starting from the left side followed the two-down-one-up rule, converging to a probability of 71% of choosing the auditory stimulus as farther to the right than the corresponding visual standard stimulus. The initial step size was $1.9^\circ$, which was decreased to $1.0^\circ$ after the first staircase reversal and again to $0.5^\circ$ after the third reversal. Each staircase comprised 36 trials. An easy trial in which the auditory test stimulus was presented at either one of the two start locations (e.g., −13.25 or $1.25^\circ$ for $-12^\circ$) was inserted once every nine trials to improve lapse-rate estimation, resulting in a total of 320 trials. The experiment was divided into four blocks. Usually, participants took about an hour and a half to complete this task.

#### Preparatory experiment 2: localization-practice task

In this task, participants practiced using the rollerball mouse to adjust the horizontal location of a response cursor to localize a stimulus. Each trial started with a central fixation cross presented for 500 ms. Then, the visual stimulus, a small white square, was displayed on the screen for 100 ms, followed by a blank screen presented for 1000 ms. Next, a response cursor appeared on the screen. Participants moved the response cursor to the location of the stimulus by moving the rollerball, and clicked the mouse to register the response. There was no time limit for the response. Visual feedback of the cursor location was provided during adjustment, but error feedback was not provided (Fig. 3A). There were eight possible horizontal positions for the stimulus, evenly spaced from −17.5 to $17.5^\circ$ in steps of $5^\circ$. Each stimulus location was visited 30 times in random order, resulting in a total of 240 trials. The inter-trial interval was 500 ms. This experiment usually took half an hour to complete.

#### Preparatory experiment 3: unimodal spatial-localization task

In this task, participants localized visual and auditory stimuli presented alone. Each trial started with a fixation cross presented straight ahead for 500 ms, followed by 1000 ms of blank screen. Then, either an auditory or a visual 100 ms-long stimulus was presented, followed by 1000 ms of blank screen. Next, the response cursor appeared, and participants adjusted the horizontal location of the cursor to match that of the stimulus. There was no time constraint for the response. Visual feedback of the cursor location was provided during its adjustment, but error feedback was not provided (Fig. 3B). After the response, the loudspeaker moved to its new location. The inter-trial interval was approximately 3000 ms.

The visual stimulus was presented at one of the four standard locations ($\pm 4$ or $\pm 12^\circ$ relative to straight ahead). Auditory stimuli were presented at the four locations that a participant perceived as spatially aligned with the four visual stimuli. These locations were identified using the data from the bimodal spatial-discrimination task (preparatory experiment 1). Each of the four locations for each modality was tested 20 times, resulting in a total of 160 trials administered in pseudorandom order. These trials were split into four blocks. Usually participants took an hour to complete this task.

#### Main experiment: bimodal spatial-localization task

The main experiment consisted of three phases: pre-learning, learning, and post-learning, and two sessions, learning congruent and incongruent stimulus statistics. In each phase, participants completed a bimodal spatial-localization task. However, the set of audiovisual stimuli differed between phases and sessions. During pre- and post-learning phases, temporally aligned audiovisual stimuli with varying spatial discrepancies were presented. During the learning phase, either only spatiotemporally aligned or only spatially and temporally discrepant audiovisual stimuli were presented. Visual and auditory stimulus locations were identified based on participants’ perceived audiovisual alignment measured using preparatory experiment 1. The combination of the four visual and four auditory stimulus locations resulted in sixteen audiovisual pairs with seven different spatial discrepancies (0, $\pm 8$, $\pm 16$, and $\pm 24^\circ$, using the perceptually equivalent visual spatial location for the auditory stimulus to compute the discrepancy) presented during the pre- and post-learning phases (Fig. 4A, left panel). For the congruent learning phase, only the audiovisual stimulus pairs with a spatial discrepancy of $0^\circ$ were presented (Fig. 4A, center panel). For the incongruent learning phase, each of the four participant-specific auditory stimulus locations was paired with a visual stimulus location either $15^\circ$ to the left or to the right of the perceptually aligned visual location. Additionally a temporal delay of $\pm 350$ ms between the onsets of the visual and auditory stimuli was introduced resulting in sixteen audiovisual stimuli with different spatiotemporal relations for the incongruent learning phase (Fig. 4A, right panel).

Each trial of the bimodal spatial-localization task started with the presentation of a centrally located fixation cross for 500 ms, followed by 1000 ms of blank screen. A 100 ms-long spatiotemporally aligned audiovisual stimulus (congruent learning phase) or a 450 ms-long spatiotemporally discrepant audiovisual stimulus (two 100 ms-long stimuli with an SOA of 350 ms; incongruent learning phase) was presented, followed by 1000 ms of blank screen. Next, the response cursor appeared. A letter located above the cursor cued which modality participants should localize (A: auditory: V: visual). After participants made their localization response, a response prompt for the unity judgment was displayed. Participants reported whether they perceived the auditory and the visual stimulus as coming from a common source or from two separate sources using the mouse keys (left-click = common source, right-click = two sources). In the learning phase, unity judgments were prompted only in 20% of randomly selected trials. After the unity judgment was given, the chosen option was highlighted for 500 ms. There was no time limit for either response, and feedback was not provided (Fig. 4A).

Each of the sixteen audiovisual stimuli presented during the pre- and post-learning phases was tested 20 times, resulting in a total of 320 trials. Each of the four stimulus pairs presented during the congruent learning phase was presented 40 times, and each of the sixteen stimulus pairs presented during the incongruent learning phase was presented ten times, resulting in a total of 160 trials for the congruent and incongruent learning phases. Trials in each phase were split into four blocks and administered in pseudorandom order. Participants completed the sequence of pre-learning, learning and post-learning phases typically in two and half hours (an hour for the pre- and post-learning phases and about half an hour for the learning phase). The order of sessions with congruent and incongruent learning phases was counterbalanced across participants.

### Data preparation and statistical analysis

#### Preparatory experiment 1: bimodal spatial-discrimination task

Responses from the bimodal spatial-discrimination task were coded as the probability of reporting the auditory test stimulus as located to the right of the visual standard stimulus. We fitted four cumulative Gaussian distributions to these data as a function of auditory stimulus location, one curve for each visual standard stimulus, with a common lapse rate, constrained to be less than 6%^65^. Adjusted $R^2$ values were calculated based on binned data (mean adjusted $R^2= 0.88$ , range 0.77–0.94, bin size = $1^\circ$). The point of subjective equality (PSE) was defined as the auditory stimulus location corresponding to a probability of 0.5 of reporting the auditory stimulus to the right of the visual one according to the psychometric function. The four PSEs were modeled as a linear function of visual stimulus location (mean $R^2= 0.97$ , range 0.92–1.00). From this linear regression of the PSEs, we computed the locations of the auditory stimuli perceived as co-located with the four visual locations. To derive error bars for the PSEs, separately for each visual standard stimulus, we randomly resampled the raw data with replacement 1,000 times, fitted psychometric curves to each resampled dataset, calculated the PSEs, and took the 2.5th and 97.5th percentiles of the 1000 PSEs as the bootstrapped confidence interval.

#### Preparatory experiment 2: localization practice task

Data from the localization practice task were not statistically analyzed but were filtered before the model fitting. We *z*-transformed the data by first subtracting the mean localization response per stimulus location and then dividing by the standard deviation of all demeaned responses. Localization responses with a *z*-score greater than 3 or smaller than −3 were identified as outliers (range 0–3.33% of trials) and excluded from model fitting.

#### Preparatory experiment 3: unimodal spatial-localization task and main experiment: bimodal spatial-localization task

Localization responses from the unimodal spatial-localization task and the bimodal spatial-localization task (pre- and post-learning phase) were compared to compute ventriloquism effects for our statistical analysis. Specifically, we first calculated the mean localization responses for each location and stimulus modality using the data from the unimodal spatial-localization task. Then, we subtracted these mean localization responses from the localization responses to the same stimulus presented at the same location but in the bimodal spatial-localization task. The differences were recoded as positive if they compensated for the spatial discrepancy between the target and the accompanying modality. These ventriloquism effects were collapsed across trials with the same absolute spatial discrepancy. To examine whether auditory and visual ventriloquism effects changed as a function of absolute spatial discrepancy between the two stimuli (computed using the perceptually equivalent visual spatial location for the auditory stimulus and converted to be an ordered factor: 0, 8, 16, $24^\circ$ to automatically test linear, quadratic, and cubic effects of the discrepancy), learning phase condition (only congruent or only incongruent stimuli), and phase (pre- and post-learning), we fitted a linear mixed-effects model (LMM) with random intercepts. Additionally, to follow up on the main effect of absolute spatial discrepancy, we conducted two-tailed one-sample *t*-tests examining whether ventriloquism effects were significantly different from zero at each absolute spatial discrepancy.

The binary unity judgments were grouped across audiovisual pairs sharing the same spatial discrepancy. We fitted a generalized linear mixed-effects model (GLMM) with binomial distribution family, logit link function, and random intercepts to individual binary responses in order to quantify the dependencies of the explicit causal-inference judgments on absolute spatial discrepancy, learning condition, and phase.

### Modeling

We begin this section by describing the three tested models and laying out the assumptions underlying each of the models, followed by the formalization of the main experiment according to the causal-inference models. Finally, we outline how each parameter was constrained by the data and describe the fitting procedure.

#### Models of the common-cause prior

We considered three nested models: (1) a high-plasticity, short-lasting changes model, (2) a high-plasticity, long-lasting changes model, and (3) a no-plasticity model. These three models differ in terms of the plasticity of the common-cause prior $p_{C=1}$ in the context of new stimulus statistics as well as the longevity of the change.

The high-plasticity, short-lasting changes model assumes that the common-cause prior can be flexibly updated after the exposure to consistently congruent or incongruent stimuli presented during the learning phase, yet, it will quickly revert to its baseline level once the observer is re-exposed to a natural environment. Therefore, this model consists of three different common-cause priors, one for the pre-learning phase of both sessions, and one for each post-learning phase:

$$\begin{aligned} \overrightarrow{p_{C=1}}= [p_{C=1,\text {pre}},p_{C=1,\text {post,cong}},p_{C=1,\text {post,incong}}]. \end{aligned}$$

The high-plasticity, long-lasting changes model is similar to the high-plasticity, short-lasting changes model; the only difference is that this model assumes changes induced during the learning phase of the first session take rather long to dissipate or might be partly revived once the participant returns to the experimental room. Therefore, this model includes an additional common-cause prior for the pre-learning phase of the second session:

$$\begin{aligned} \overrightarrow{p_{C=1}} = [p_{C=1,\text {pre,cong}},p_{C=1,\text {post,cong}},p_{C=1,\text {pre,incong}},p_{C=1,\text {post,incong}}]. \end{aligned}$$

These two models were contrasted with a no-plasticity model. This model assumes that the common-cause prior remains unchanged after the exposure to consistently congruent or incongruent audiovisual stimuli during the learning phase:

$$\begin{aligned} \overrightarrow{p_{C=1}} = [p_{C=1}]. \end{aligned}$$

#### Model assumptions

A stimulus presented at location *s* leads to a sensory measurement in the observer’s brain. This measurement is corrupted by Gaussian noise. With repeated presentation of an identical stimulus at location *s*, the measurements correspond to scattered spatial locations $m' \sim {\mathcal {N}}(s',\sigma ')$, with standard deviation $\sigma '$. To allow integration of information from different modalities, measurements from the different modalities are remapped into a common internal reference frame. Hence, the measurement distribution is centered at a remapped location, $s_A'=a_A s_A + b_A$ given auditory location $s_A$, and $s_V'=a_V s_V + b_V$ given visual location $s_V$.

Participants have prior knowledge about the typical location of stimuli. The probability distribution across locations is assumed to be a Gaussian distribution centered at $\mu _P'$ with standard deviation $\sigma _P'$, i.e., $s'\sim {\mathcal {N}}(\mu _P', \sigma _P')$. This spatial prior is assumed to be in units of the common internal reference frame. This spatial prior distribution is assumed to be modality-independent, i.e., to hold for all three stimulus types presented in this study (unimodal auditory stimuli at location $s_A$, unimodal visual stimuli at location $s_V$, and bimodal audiovisual stimuli at locations $s_{AV}$). Fitting the bias parameters $a_A$, $b_A$, $a_V$, $b_V$, and the parameters for the supra-modal prior $\mu _P'$ and $\sigma _P'$ at once is impossible as they effectively trade off. Thus, we set the remapped location $s_V'$ equal to the physical location $s_V$ (i.e., $a_V = 1$, $b_V=0$), and assumed a supra-modal central prior (i.e., $\mu _P'=0$).

We assume that the measurement noise differs between unimodal and bimodal stimuli^8,21,66,67^. We denote $\sigma _{A}'$ and $\sigma _{V}'$ as the measurement noise when an auditory or a visual stimulus is presented unimodally as in the bimodal spatial-discrimination and unimodal spatial-localization tasks, and $\sigma _{AV,A}'$ and $\sigma _{AV,V}'$ for the bimodal auditory and visual stimuli presented during the pre- and post-learning phases of the main experiment.

#### Formalization of the main experiment

During the pre- and post-learning phases of the main experiment, the observer was presented with an audiovisual stimulus pair $AV_l$, which consisted of an auditory stimulus at location $s_{{AV_l},A}$ and a visual stimulus at location $s_{{AV_l},V}$. Combining the four visual and the four auditory stimulus locations resulted in 16 different audiovisual stimulus pairs ($l\in \{1,2,\ldots ,16\}$). The presentation of an audiovisual stimulus pair leads to two noisy sensory measurements in the observer’s brain, one for the auditory, $m_{AV_l,A}'\sim {\mathcal {N}}(s_{AV_l,A}',{\sigma _{AV,A}'})$ $={\mathcal {N}}(a_As_{AV_l,A} + b_A,{\sigma _{AV,A}'})$, and the other for the visual stimulus, $m_{AV_l,V}'\sim {\mathcal {N}}(s_{AV_l,V}',{\sigma _{AV,V}'})={\mathcal {N}}(s_{AV_l,V},{\sigma _{AV,V}'})$.

Based on the sensory measurements, the observer derives two location estimates, one for the common-cause scenario, $C=1$, and the other for the separate-causes scenario, $C=2$. In the common-cause scenario, the location estimate of the auditory element ${\hat{s}}_{AV_l, A,C=1}'$ is the same as that of the visual element ${\hat{s}}_{AV_l, V,C=1}'$, which equals the reliability-weighted average of the sensory measurements $m_{AV_l,A}'$, $m_{AV_l,V}'$, and the mean $\mu _P'$ of the spatial supra-modal prior, which has variance ${\sigma _P'}^2$:

1 $$\begin{aligned} {\hat{s}}_{AV_l, A,C=1}' = {\hat{s}}_{AV_l, V,C=1}'=\frac{m_{AV_l, A}'\sigma _{AV,A}^{-2} + m_{AV_l, V}'\sigma _{AV,V}^{-2} + \mu _P' {\sigma _P'}^{-2}}{\sigma _{AV,A}^{-2}+\sigma _{AV,V}^{-2} + {\sigma _P'}^{-2}}. \end{aligned}$$

In the separate-causes scenario, the location estimate of the auditory stimulus, ${\hat{s}}_{AV_l, A,C=2}'$, is equal to the reliability-weighted average of the auditory measurement $m_{AV_l,A}'$ and the mean of the spatial prior $\mu _P'$:

2 $$\begin{aligned} {\hat{s}}_{AV_l, A,C=2}' =\frac{m_{AV_l, A}'\sigma _{AV,A}^{-2} + \mu _P' {\sigma _P'}^{-2}}{\sigma _{AV,A}^{-2}+ {\sigma _P'}^{-2}}. \end{aligned}$$

Analogously, the location estimate of the visual stimulus, ${\hat{s}}_{AV_l, V,C=2}'$, is equal to the reliability-based average of the visual measurement $m_{AV_l,V}'$ and the mean of the spatial prior $\mu _P'$:

3 $$\begin{aligned} {\hat{s}}_{AV_l, V,C=2}' =\frac{m_{AV_l, V}'\sigma _{AV,V}^{-2} + \mu _P' {\sigma _P'}^{-2}}{\sigma _{AV,V}^{-2}+ {\sigma _P'}^{-2}}. \end{aligned}$$

The final location estimates are derived by model averaging (see Appendix S5.1 for model selection, an alternative decision strategy that performed less well). Specifically, the final auditory location estimate ${\hat{s}}_{AV_l,A}'$ is the average of the two intermediate auditory location estimates, the one for the common-cause scenario and the other for the separate-causes scenario, with each weighted by the posterior probability of the corresponding causal structure^17,28^:

4 $$\begin{aligned} {\hat{s}}'_{AV_l,A}={\hat{s}}'_{AV_l,A,C=1}P(C=1|m'_{AV_l,A},m'_{AV_l,V})+{\hat{s}}'_{AV_l,A,C=2}\left( 1-P(C=1|m'_{AV_l,A},m'_{AV_l,V})\right) , \end{aligned}$$

and analogously for the final location estimate ${\hat{s}}_{AV_l,V}'$:

5 $$\begin{aligned} {\hat{s}}'_{AV_l,V}={\hat{s}}'_{AV_l,V,C=1}P(C=1|m'_{AV_l,A},m'_{AV_l,V})+{\hat{s}}'_{AV_l,V,C=2}\left( 1-P(C=1|m'_{AV_l,A},m'_{AV_l,V})\right) . \end{aligned}$$

The posterior probability of a common source for the auditory and visual measurements is proportional to the product of the likelihood of a common source for the two measurements and the common-cause prior $p_{C=1}$:

6 $$\begin{aligned} P(C=1|m'_{AV_l,A},m'_{AV_l,V})=\frac{P(m'_{AV_l,A},m'_{AV_l,V}|C=1)p_{C=1}}{P(m'_{AV_l,A},m'_{AV_l,V}|C=1)p_{C=1}+P(m'_{AV_l,A},m'_{AV_l,V}|C=2)(1-p_{C=1})}. \end{aligned}$$

The posterior probability of two separate sources is $1-P(C=1|m'_{AV_l,A},m'_{AV_l,V})$. The likelihood of a common source given the auditory and visual measurements, $P(m'_{AV_l,A},m'_{AV_l,V}|C=1)$, is the product of the likelihood of the internally represented audiovisual stimulus location $s_{AV_l}'$ given the auditory and visual measurements, and the supra-modal prior, integrated over all possible values of $s_{AV_l}'$^17^:

7 $$\begin{aligned} \begin{aligned}{}&P(m'_{AV_l,A},m'_{AV_l,V}|C=1) =\int P(m'_{AV_l,A}|s_{AV_l}')P(m'_{AV_l,V}|s_{AV_l}')P(s_{AV_l}') ds_{AV_l}'\\&\quad =\frac{1}{2\pi \sqrt{{\sigma _{AV,A}'}^2 {\sigma _{AV,V}'}^2 + {\sigma _{AV,A}'}^2 {\sigma _P'}^2 + {\sigma _{AV,V}'}^2 {\sigma _P'}^2}} \times \exp \Bigg [-\frac{1}{2}\frac{(m_{AV_l,A}' - m_{AV_l,V}')^2 {\sigma _P'}^2 + (m_{AV_l,A}' - \mu _P')^2 {\sigma _{AV,V}'}^2 + (m_{AV_l,V}' - \mu _P')^2 {\sigma _{AV,A}'}^2}{{\sigma _{AV,A}'}^2 {\sigma _{AV,V}'}^2 + {\sigma _{AV,A}'}^2 {\sigma _{P'}^2} + {\sigma _{AV,V}'}^2{\sigma _{P'}^2}} \Bigg ] . \end{aligned} \end{aligned}$$

The likelihood of different sources given the two measurements, $P(m'_{AV_l,A},m'_{AV_l,V}|C=2)$ is the product of the likelihood of internally represented auditory and visual stimulus location $s_A'$ and $s_V'$, and the supra-modal prior. Given that the measurements in this causal scenario stem from different sources, the product is integrated over all possible, remapped visual and auditory stimulus locations, $s_A'$ and $s_V'$:

8 $$\begin{aligned} \begin{aligned}{}&P(m_{AV_l,A}',m_{AV_l,V}'|C=2) = \int \int P(m_{AV_l,A}',m_{AV_l,V}'|s_{AV_l,A}', s_{AV_l,V}')P(s_{AV_l,A}', s_{AV_l,V}') ds_{AV_l,A}' ds_{AV_l,V}' \\&\quad =\left( \int P(m_{AV_l,A}'|s_{AV_l,A}') P(s_{AV_l,A}') ds_{AV_l,A}'\right) \left( \int P(m_{AV_l,V}'|s_{AV_l,V}') P(s_{AV_l,V}') ds_{AV_l,V}'\right) \\&\quad =\frac{1}{2\pi \sqrt{({\sigma _{AV,A}'}^2 + {\sigma _P'}^2)({\sigma _{AV,V}'}^2 + {\sigma _P'}^2)}} \exp \Bigg [-\frac{1}{2} \left( \frac{(m_{AV_l,A}'-\mu _P')^2}{{\sigma _{AV_l,A}'}^2 +{\sigma _P'}^2} + \frac{(m_{AV_l,V}'-\mu _P')^2}{{\sigma _{AV_l,V}'}^2 +{\sigma _P'}^2}\right) \Bigg ]. \end{aligned} \end{aligned}$$

For the localization responses, we assume that the probability distribution of the localization response $r_{AV_l,A}$ has a Gaussian shape, centered on the final location estimate ${\hat{s}}_{AV_l,A}'$ with perception-unrelated noise variance $\sigma _r^2$:

9 $$\begin{aligned} P(r_{AV_l,A}|{\hat{s}}_{AV_l,A}')=\phi (r_{AV_l,A}; {\hat{s}}_{AV_l,A}', \sigma _r), \end{aligned}$$

where $\phi$ is the Gaussian density function. Analogously, the probability distribution of the localization responses $r_{AV_l,V}$ is

10 $$\begin{aligned} P(r_{AV_l,V}|{\hat{s}}_{AV_l,V}')=\phi (r_{AV_l,V}; {\hat{s}}_{AV_l,V}', \sigma _r). \end{aligned}$$

For the unity judgments, we assume that observers adopted a heuristic decision rule, which is based on the distance between the final auditory and visual location estimates, ${\hat{s}}_{AV_l,A}'$ and ${\hat{s}}_{AV_l,V}'$. If the distance between them is below an internal criterion $\varepsilon$, then the observer intends to report a common cause^8^. Otherwise, the observer intends to report separate causes (see Appendix S5.2–5.3 for an optimal, common-cause-posterior-based and a measurements-based decision strategy that performed less well). However, due to occasional lapses ($\lambda _{\text {unity}}$), the observer might mistakenly report separate causes even when the intended response is a common cause,

11 $$\begin{aligned} P(I_{C=1}=1|m'_{AV_l,A},m'_{AV_l,V}) ={\left\{ \begin{array}{ll} 1-\lambda _{\text {unity}} &{}\text {if } |{\hat{s}}_{AV_l, A}' -{\hat{s}}_{AV_l, V}'|<\varepsilon \\ \lambda _{\text {unity}} &{}\text {otherwise}.\\ \end{array}\right. } \end{aligned}$$

#### Model fitting

For a given model *M*, we fitted the model jointly to the data from the bimodal spatial-discrimination task, the localization responses from the unimodal spatial-localization task, and the localization responses and unity judgments from the bimodal spatial-localization task during the pre- and post-learning phases in both the congruent and incongruent conditions. The localization responses from the localization practice task were fitted separately to reduce the number of free parameters estimated simultaneously. The data from the learning phase were excluded as they are sequentially dependent, making them computationally challenging to fit. All models were fit using a maximum-likelihood procedure (see Appendix S5.4 for log-likelihoods of all models). Next, we describe model parameters and how they are constrained by different tasks.

##### Sensory noise for bimodal trials, common-cause priors, and lapse rates

The log-likelihood of these parameters depends on the localization responses, $r_{AV_l,A}$ and $r_{AV_l,V}$, as well as the unity judgment, $I_{C=1}$. According to the causal inference model, both of them depend in turn on the measurement noise variances ${\sigma _{AV,A}'}^2$ and ${\sigma _{AV,V}'}^2$ under bimodal presentation mode and the variance of the supra-modal prior distribution ${\sigma_P'}^2$, as these variances influence the location estimates via the weighting of the two measurements and the central prior (Eq. 3) and via the likelihood of a common cause (Eq. 7). The location estimates also depend on the posterior probability of a common cause (Eq. 6), which in turn depends on the set of common-cause priors $\overrightarrow{p_{C=1}}$. Finally, the unity judgements depend on the two lapse rates $\lambda _{\text{unity}}$ (one for each condition). These free parameters were constrained solely by data from the pre- and post-learning phases of the main experiment (Table 2).

**Table 2:** Summary of model parameters and parameter estimation.

Parameter	Meaning	Constrained by			
		Pre-expt.1	Pre-expt.2	Pre-expt.3	Main expt.
$a_A$	$\text {The proportional bias in auditory spatial} \text { perception}$	$\checkmark$	–	$\checkmark$	$\checkmark$
$b_A$	$\text {The constant bias in auditory spatial perception}$	$\checkmark$	–	$\checkmark$	$\checkmark$
${\sigma _A'}^2$	$\text {Measurement noise variance for the auditory} \text {stimulus in unimodal trials}$	$\checkmark$	–	$\checkmark$	–
${\sigma _V'}^2$	$\text {Measurement noise variance for the visual } \text {stimulus in unimodal trials}$	$\checkmark$	–	$\checkmark$	–
$\lambda _{AV}$	$\text {Lapse rate for pre-expt. 1}$	$\checkmark$	–	–	–
$\sigma _r^2$	$\text {Variance of the response distribution for a } \text {maximally reliable visual stimulus}$	–	$\checkmark$	–	–
${\sigma _{AV,A}'}^2$	$\text {Measurement noise variance for the auditory } \text {stimulus in bimodal trials}$	–	–	–	$\checkmark$
${\sigma _P'}^2$	$\text {The variance of the supra-modal prior over } \text {stimulus location}$	$\checkmark$	–	$\checkmark$	$\checkmark$
${\sigma _{AV,V}'}^2$	$\text {Measurement noise variance for the visual } \text { stimulus in bimodal trials}$	–	–	–	$\checkmark$
$\overrightarrow{p_{C=1}}$	$\text {Common-cause priors}$	–	–	–	$\checkmark$
$\varepsilon$	The internal criterion for making unity judgments	–	–	–	$\checkmark$
$\lambda _{\text {unity, cong}}$	$\text {Lapse rate for the unity judgments in the session } \text {where the exposure stimuli were congruent}$	–	–	–	$\checkmark$
$\lambda _{\text {unity, incong}}$	$\text {Lapse rate for the unity judgments in the session } \text {where the exposure stimuli were incongruent}$	–	–	–	$\checkmark$

##### Motor/memory noise

The localization responses, $r_{AV_l,A}$ and $r_{AV_l,V}$, additionally depend on the variability in responses due to factors other than perceptual noise, for example, loss of precision in memory or the use of the response device, $\sigma _r^2$. This parameter was constrained solely by the localization practice task (Table 2). Since we used visual stimuli for which participants’ localization was highly precise in the preparatory experiment, the effect of the supra-modal spatial prior on the final visual location estimate $r_V$ should be negligible, and consequently the probability distribution of the localization responses is centered on the physical visual stimulus location $s_V$. Furthermore, since the response noise should be due to multiple, uncorrelated perception-unrelated sources of variability, we assume the probability distribution of the localization responses is Gaussian. With these assumptions, the value of $\sigma _r^2$ that maximizes the likelihood can be calculated in closed form, that is,

12 $$\begin{aligned} \sigma _r^2 =\bigg [\sum _{n=1}^{N}(r_{V,n}-s_{V,n})^2\bigg ]/N, \end{aligned}$$

where *n* indexes the trials in the localization practice task, and *N* denotes the total number of trials excluding outliers.

##### Perceptual biases, sensory noise for unimodal trials and lapse rates

The localization responses, $r_{AV_l,A}$ and $r_{AV_l,V}$, as well as the unity judgment, $I_{C=1}$, also depend on the bias parameters $a_A$ and $b_A$ (Eqs. 9 and 10). In addition to the main experiment, these bias parameters were also constrained by the unimodal spatial-localization task and the bimodal spatial-discrimination task (Table 2). Specifically, in the unimodal spatial-localization task, participants localized either a unimodal visual or a unimodal auditory stimulus in each trial. The localization responses are centered at the location estimates $\mu _{{\hat{s}}'}$ in perceptual space. The variability of the localization responses consists of perception-related variability $\sigma _{{\hat{s}}'}^2$ and perception-unrelated variability $\sigma _r^2$. In other words, the probability distributions of the localization responses are

13 $$\begin{aligned} r_{A,u}\sim {\mathcal {N}}\Big (\mu _{{\hat{s}}_{A,u}}, \sqrt{\sigma _{{\hat{s}}_A'}^2 + \sigma _r^2}\Big ) \text { and } r_{V,w}\sim {\mathcal {N}}\Big (\mu _{{\hat{s}}_{V,w}}, \sqrt{\sigma _{{\hat{s}}_V'}^2 + \sigma _r^2}\Big ), \end{aligned}$$

where *u* indexes the participant-specific auditory stimulus location, and *w* indexes the visual stimulus location ($s_V\in \{-12,-4,4,12^\circ \}$). The center of the localization responses $\mu _{{\hat{s}}'}$ relates the physical stimulus location to the internal location estimates via the bias parameters $a_A$ and $b_A$ (see Appendix S6.1 for the derivation):

14 $$\begin{aligned} \mu _{{\hat{s}}_{A,u}} = \frac{(a_A s_{A,u} + b_A){\sigma _A'}^{-2} + \mu _P' {\sigma _P'}^{-2}}{{\sigma _A'}^{-2} + {\sigma _P'}^{-2}} \text { and } \mu _{{\hat{s}}_{V,w}} =\frac{s_{V,w}{\sigma _V'}^{-2} + \mu _P' {\sigma _P'}^{-2}}{{\sigma _V'}^{-2} + {\sigma _P'}^{-2}}. \end{aligned}$$

In the bimodal spatial-discrimination task participants were presented with a visual standard and an auditory test stimulus in a random order in each trial, and they reported whether the perceived auditory stimulus location $s_A$ was to the right or left of the visual standard stimulus $s_V$. The binary judgments were then recoded as the proportion of reporting the auditory stimulus to the right of the visual standard stimulus $p_{I_{\text {A-right}}=1}$, which in turn was modeled as a cumulative Gaussian distribution with a lapse rate $\lambda _{AV}$:

15 $$\begin{aligned} p_{I_{\text {A-right},w,o}=1} = 0.5\lambda _{AV} + (1-\lambda _{AV})\Big [1-\Phi \Big (0 ;\mu _{{\hat{s}}'_{A,o}} - \mu _{{\hat{s}}'_{V,w}}\Big ), \sigma _{{\hat{s}}'_{A}}^2 + \sigma _{{\hat{s}}'_{V}}^2\Big ], \end{aligned}$$

where *o* indexes the finer grid of auditory locations (see Appendix S6.2 for a derivation).

As described in Eqs. (12)–(14), the variabilities of perceived unimodal stimuli, $\sigma _{{\hat{s}}_A'}^2$ and $\sigma _{{\hat{s}}_V'}^2$, are constrained by both the unimodal spatial-localization task and bimodal spatial-discrimination task (Table 2). They relate to the variance of the measurement noise in unimodal trials as follows (see Appendix S6.1 for derivation):

16 $$\begin{aligned} \sigma _{{\hat{s}}_A'}^2=\Big (\frac{{\sigma _A'}^{-2}}{{\sigma _A'}^{-2} + {\sigma _P'}^{-2}}\Big )^2 {\sigma _A'}^2 \text { and } \sigma _{{\hat{s}}_V'}^2 = \Big (\frac{{\sigma _V'}^{-2}}{{\sigma _V'}^{-2} + {\sigma _P'}^{-2}}\Big )^2 {\sigma _V'}^2. \end{aligned}$$

#### Parameter estimation

For each model *M*, we searched for the combination of model parameters that maximized the log likelihood using the BADS toolbox^68^. To deal with the possibility that the returned parameter values might correspond to a local minimum, we ran BADS multiple times with 20 different starting points, randomly chosen from a $D_M$-dimensional space, where $D_M$ is the number of free parameters for model *M*. The final parameter estimates were those with the maximum likelihood across all runs of the fitting procedure (see Appendix S7 for estimated parameters and log likelihood of the best-fitting model for all participants).

To derive error bars for the common-cause priors, we selected the best-fitting model for each participant (see “Model comparison”), and fitted it to 120 bootstrapped datasets, resampled separately for each experiment. The fitting procedure given each bootstrapped dataset was the same as just described. At the end, we took the 2.5th and 97.5th percentiles of the 120 common-cause priors as the bootstrapped confidence interval.

#### Model comparison

To compare model performance quantitatively, we computed the Akaike information criterion (AIC)^69^ for all models, separately for each participant. The best-fitting model corresponds to the model with the minimal AIC value. We then computed relative model-comparison scores, $\Delta _{AIC}$, which describes the AIC value of each model relative to that of the best-fitting model. The best-fitting model has a $\Delta _{AIC}$ value of 0, and a high $\Delta _{AIC}$ value for other models indicates stronger evidence for the best-fitting model. We counted the number of times the high-plasticity, short-lasting changes, the high-plasticity, long-lasting changes, and the no-plasticity model won across participants, with a conservative criterion (there is only one best model, the one with the minimal AIC value) and a liberal criterion (all models with an AIC value exceeding the minimal AIC value by less than 2 are counted as winner model; see Appendix S8 for a comparison of all candidate models).

#### Model predictions

We predicted ventriloquism effects and proportions of reporting a ‘common cause’ for each participant based on the best-fitting model and parameters. Specifically, to compute the model-predicted ventriloquism effects, we calculated the difference between the expected value regarding the auditory/visual localization response for any given audiovisual pair and the mean perceived location when the auditory/visual element of the pair was presented alone (see Appendix S9.1). The difference was then averaged across audiovisual stimulus pairs with the same absolute spatial discrepancy. To compute the model-predicted proportion of reporting a ‘common cause’ for a given audiovisual pair, we summed, across all possible combinations of auditory and visual measurements, the probability of reporting a ‘common cause’, weighted by the joint probability of the corresponding auditory and visual measurements (see Appendix S9.2). The proportion of reporting a ‘common cause’ was then averaged across audiovisual stimulus pairs with the same absolute spatial discrepancy.

To add error bars to the model predictions, we used parametric bootstrapping. Specifically, we drew a set of samples, 1000 times, from the model-predicted probability distributions for bimodal and unimodal localization responses. The number of samples in each set matched the number of trials in our study. Given the samples, we computed ventriloquism effects as a function of absolute spatial discrepancy for each simulation run, sorted them in ascending order, and computed the 2.5 and 97.5% percentiles as the 95% confidence interval (see Appendix S9.3). Similarly, we drew binary unity judgments from the model-predicted proportion of reporting a ’common cause’. Given the samples, we computed proportions of reporting a ‘common cause’ as a function of absolute spatial discrepancy, sorted them in ascending order, and computed the 2.5 and 97.5% percentiles as the 95% confidence interval (see Appendix S9.4).

### Simulations

First, we examined via simulation the effects of the variability of auditory and visual location measurements and the distance between the two measurements on the posterior probability of a common cause (Fig. 6A,B; Eq. 6). These variables influence the posterior probability of a common cause via the conditional location estimate given the common-cause scenario (Eq. 3) as well as the likelihood of a common cause^17^. In each simulation, we either varied the measurement noise or the discrepancy between the auditory and visual stimuli (see Appendix S10.1 for parameter values). For each audiovisual stimulus pair, we simulated 10,000 pairs of auditory and visual measurements and computed the posterior probability of a common cause using Eq. (4).

We explored the driving factors that could lead to either increases or decreases of the common-cause prior by simulating its updates during the learning phase. Specifically, we assumed a fixed value of the common-cause prior at the beginning of the learning phase equal to the one estimated based on the data from the pre-learning phase, that is $p_{C=1}(t=1)= p_{C=1,pre}$. For each trial, the simulated observer was presented with an audiovisual stimulus pair, leading to two noisy measurements. The observer then derived the posterior probabilities of each causal scenario. The common-cause prior for the next trial was shifted in the direction of the posterior probability of a common cause for the current trial, that is,

17 $$\begin{aligned} p_{C=1}(t+1) = p_{C=1}(t) + \alpha _{p_{C=1}}\left( p(C=1|m_{AV,A}'(t), m_{AV,V}'(t)) - p_{C=1}(t)\right), \end{aligned}$$

where $\alpha _{p_{C=1}}$ governs the size of the update relative to the discrepancy between the current common-cause prior and the posterior of a common cause in the current trial. The choice of $\alpha _{p_{C=1}}$ influenced the size of the modulation but not its direction. The modulation of the common-cause prior is defined as the difference between the final common-cause prior $p_{C=1}(t=161)$, after 160 learning trials as in our experiment, and the prior at the beginning of the learning phase $p_{C=1}(t=1)$. Each simulation was repeated 100 times, and these simulations were run for many combinations of the variabilities of the auditory and visual measurements as well as, discrepancies between the measurements (see Appendix S10.2 for parameter values), given different ‘startup’ common-cause prior at the beginning of the learning phase (see Appendix S10.3).

## Supplementary Information

Supplementary Information.

## Data Availability

All data and code files are available on an OSF archive (https://osf.io/amrp7/).
