# Supplementary material for: Repeated exposure to either consistently spatiotemporally congruent or consistently incongruent audiovisual stimuli modulates the audiovisual common-cause prior
Source: Sci Rep. 2022 Sep 15;12:15532. doi: 10.1038/s41598-022-19041-7 (PMC9478143; doi:10.1038/s41598-022-19041-7)
Supplement: Supplementary file 1 — Supplementary Information. [file 41598_2022_19041_MOESM1_ESM.pdf]

Appendix: Repeated exposure to either consistently spatiotemporally congruent or consistently incongruent audiovisual stimuli modulates audiovisual common-cause prior

Fangfang Hong[1,*], Stephanie Badde[2+], and Michael S. Landy[1,3+]

[1]Psychology department, New York University, New York, NY, USA

[2]Psychology department, Tufts University, Medford, MA, USA

[3]Center for Neural Science, New York University, New York, NY, USA

[*]Corresponding author email: fangfang.hong@nyu.edu

[+]Equal contributions

Contents

S1 Results for the bimodal spatial-discrimination task **3**

S2 Excluding outlier participants **4**

S3 Statistical analysis **5**
- S3.1 Auditory ventriloquism effects . . . 5
- S3.2 Visual ventriloquism effects . . . 6
- S3.3 Unity judgments . . . 7

S4 Individual-level empirical data and model predictions **9**
- S4.1 Participants whose common-cause prior about vision and audition was updated in the direction of the new stimulus statistics . . . 9
- S4.2 Participants whose spatial common-cause prior about vision and audition was updated in the opposite direction of stimulus statistics . . . 10

S5 Modeling **11**
- S5.1 An alternative decision strategy for implicit causal inference - model selection . . . 11
- S5.2 Alternative decision strategies for explicit causal inference . . . 11
  - S5.2.1 Optimal posterior-based unity judgments . . . 11
  - S5.2.2 measurement-based (heuristic) unity judgments . . . 11
- S5.3 Overview of models with different decision strategies and assumption about the plasticity of the common-cause prior . . . 12
- S5.4 Model likelihoods . . . 12
  - S5.4.1 Model likelihoods given the bimodal spatial-discrimination task . . . 12
  - S5.4.2 Model likelihoods given unimodal localization responses . . . 13
  - S5.4.3 Model likelihoods given bimodal spatial-localization responses and unity judgments . . . 13
  - S5.4.4 Model likelihoods of models that assume different decision strategies . . . 14

S6 Mathematical derivations **16**
- S6.1 The center and the spread of the distribution for localization responses . . . 16
- S6.2 The psychometric functions for the bimodal spatial-discrimination task . . . 16

S7 Estimated parameters given the best-fitting model **18**
- S7.1 All relevant free model parameters . . . 18
- S7.2 Best-fitting models . . . 19
- S7.3 Estimated common-cause priors given the best-fitting model . . . 19

S8 Complete model comparison results **21**

S9 Model predictions and parametric bootstrapping **22**
- S9.1 Predicted probability distributions for localization responses . . . 22
- S9.2 Predicted proportion of reporting ‘common cause’ . . . 23
- S9.3 Confidence intervals for model-predicted ventriloquism effects using parametric bootstrapping . . . 23
- S9.4 Confidence intervals for model-predicted proportions of reporting ‘common cause’ using parametric bootstrapping . . . 23

S10 Simulated posterior probabilities of a common cause and changes of the common-cause prior given varying sensory measurement noise **25**
S10.1 The posterior probability of a common cause 25
S10.2 The updates of the common-cause prior 25
S10.3 The effects of the 'startup' common-cause prior on its updates 27

References **28**

S1 Results for the bimodal spatial-discrimination task

In the first preparatory experiment, we measured participants' modality-specific biases in auditory relative to visual spatial perception. Specifically, participants determined whether an auditory test stimulus, the location of which varied within a wide range, was to the left or right of a visual standard stimulus, the location of which was predetermined (±4° or ±12°). We fitted four cumulative Gaussian distributions to the binary judgments as a function of auditory stimulus location, one for each visual stimulus location, with a common lapse rate, which was constrained to be less than 6%[1]. For each psychometric function, we calculated the PSE, and fitted a linear regression line to those four PSEs as a function of visual stimulus location (Fig. 2B, right panel). The estimated slopes for all participants were significantly smaller than 1 (mean = 0.593°, SEM = 0.042°, Fig. S1). Thus, the auditory stimuli were perceived as shifted towards the periphery relative to the visual stimuli, which is in line with previous findings[2–5]. Ten out of 17 participants (outliers excluded; see S2 Excluding outlier participants) showed significant positive intercepts (mean = 2.106°, SEM = 0.469°, Fig. S1), indicating that they perceived the auditory stimuli as shifted to the left relative to visual stimuli.

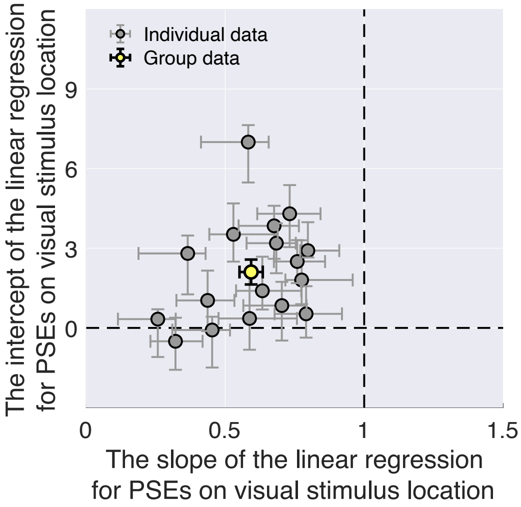

Figure S1. Results for the bimodal spatial-discrimination task. The slope (x-axis) and the intercept (y-axis) of the linear regression for PSEs on visual stimulus location. Grey circles: individual data; yellow circle: group mean; grey error bars: 95% bootstrapped confidence intervals; black error bars: standard error of the mean (SEM). Vertical and horizontal dashed lines correspond to the absence of proportional and constant perceptual biases of audition relative to vision, respectively.

S2 Excluding outlier participants

In the unimodal spatial-localization task (preparatory experiment 3), participants localized either unimodal visual stimulus, presented at either ±12° or ±4°, or unimodal auditory stimuli, the locations of which were identified individually for each participant in the bimodal spatial-localization task (Preparatory Experiment 1). The localization responses from this experiment enabled us to directly measure modality-specific biases, and therefore, provided us a chance to re-evaluate whether the participant-specific auditory stimulus locations truly matched the four visual stimulus locations. To this aim, we computed the mean auditory and visual localization responses (Fig. S2A), and fitted two linear regressions, one for the mean auditory responses and the other for the mean visual responses, on visual stimulus locations (Fig. S2B). The slope of the two linear regression, which reflects the proportional bias in spatial perception, should match if the participant-specific auditory stimulus locations were perceived as co-located with the four pre-selected visual stimulus locations; the same holds true for the intercept, which reflects the constant bias in spatial perception. Therefore, we computed the mean absolute value of the difference between the slopes (M = 0.313°, SD = 0.246°), and the intercepts (M = 2.149°, SD = 1.641°). We excluded participants who showed a difference of the slopes or the intercepts greater than 3 SDs or smaller than -3 SDs. Three participant met these criteria, and thus were excluded from further analysis and model fitting (Fig. S2C). With these outliers excluded, the mean absolute value of the difference between slopes was reduced (M = 0.243°, SD = 0.169°) as was the difference between intercepts (M = 1.965°, SD = 1.053°).

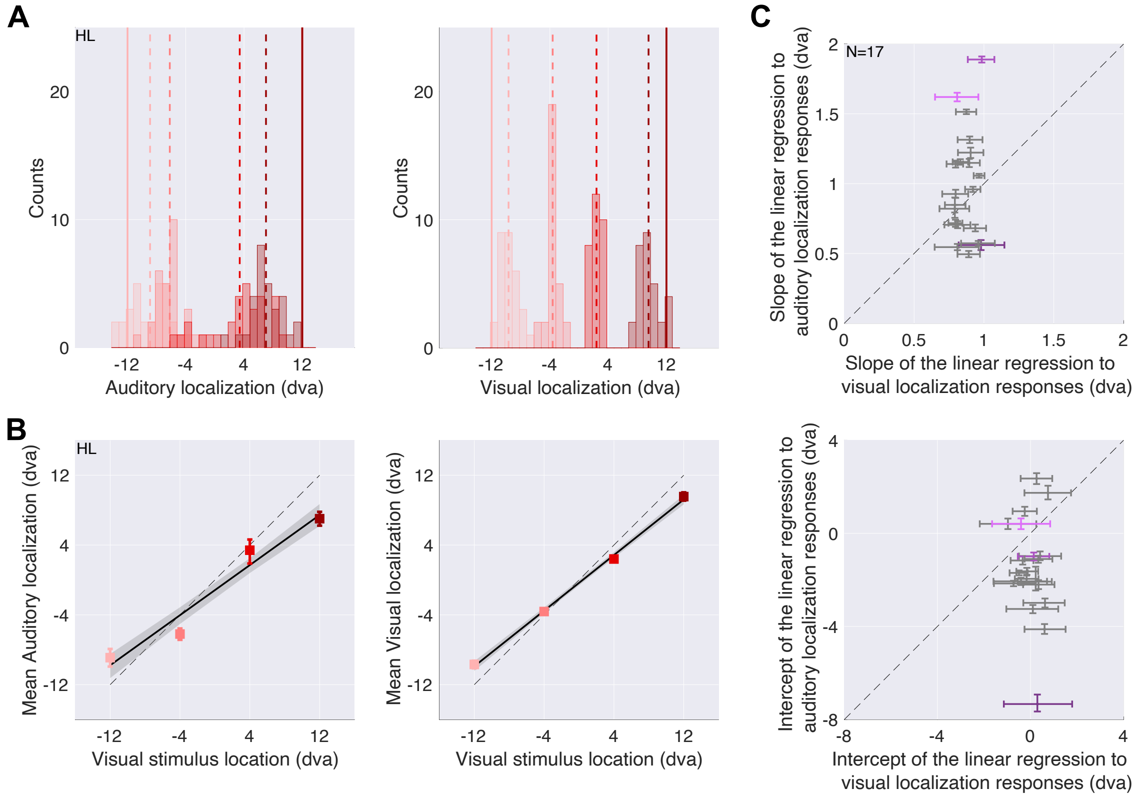

Figure S2. Results from the unimodal spatial-localization task (preparatory experiment 2). (A) Histograms for unimodal auditory (left panel) and unimodal visual (right panel) localization responses for a representative participant HL. Solid lines: the four pre-selected visual stimulus locations; dashed lines: mean localization responses; shades of pink: stimulus locations from left (light pink) to right (dark pink). (B) Linear regression fit to the mean auditory (left panel) and visual (right panel) localization responses on visual stimulus location. Error bars: 95% bootstrapped confidence intervals; grey areas: linear regressions based on all bootstrapped datasets. (C) The slope (upper panel) and the intercept (lower panel) of the linear regression of the mean visual localization responses on visual stimulus location plotted against those of the mean auditory localization responses on visual stimulus location. Error bars: 95% bootstrapped confidence intervals; diagonal dashed lines: identity line. Grey data points: non-outlier participants; purple data points: identified outlier participants.

S3 Statistical analysis

S3.1 Auditory ventriloquism effects

Table S1. Linear mixed-effects model (LMM) analysis for the auditory ventriloquism effects from the pre- and post-learning phases of the main experiment. Test statistics reveal whether the estimated beta is significantly different from 0 (greater absolute t-value indicates that β is further away from 0) and the results of model comparison between the null and alternative models (larger χ^2 indicates stronger evidence for the alternative models).

	Estimated β (Confidence interval)	Test statistics and p-value	
		Significance of β compared to 0 (t-value)	Model comparson
Intercept	4.24 (5.93, 6.09)	8.11	-
$\lvert s_V - s_A \rvert$	3.32 (2.78, 3.86)	12.03	$\chi^2(3) = 891.94$, $p < 0.001 ***$
$\lvert s_V - s_A \rvert^2$	-0.92 (-1.40, -0.43)	-3.68	
$\lvert s_V - s_A \rvert^3$	0.25 (-0.18, 0.67)	1.12	
Condition	-0.18 (-0.52, 0.17)	-1.00	$\chi^2(1) = 0.00$, $p = 0.95$
Phase	0.01 (-0.33, 0.36)	0.07	$\chi^2(1) = 2.42$, $p = 0.12$
Condition:phase	0.25 (-0.23, 0.74)	1.02	$\chi^2(1) = 2.20$, $p = 0.14$
$\lvert s_V - s_A \rvert$:condition	0.33 (-0.44, 1.09)	0.84	$\chi^2(3) = 5.13$, $p = 0.16$
$\lvert s_V - s_A \rvert^2$:condition	-0.31 (-1.00, 0.38)	-0.88	
$\lvert s_V - s_A \rvert^3$:condition	0.16 (-0.45, 0.76)	0.51	
$\lvert s_V - s_A \rvert$:phase	0.19 (-0.57, 0.96)	0.50	$\chi^2(3) = 1.70$, $p = 0.74$
$\lvert s_V - s_A \rvert^2$:phase	-0.18 (-0.88, 0.51)	-0.53	
$\lvert s_V - s_A \rvert^3$:phase	-0.08 (-0.69, 0.52)	-0.27	
$\lvert s_V - s_A \rvert$:condition:phase	-0.46 (-1.54, 0.62)	-0.84	$\chi^2(3) = 0.93$, $p = 0.82$
$\lvert s_V - s_A \rvert^2$:condition:phase	-0.27 (-1.25, 0.70)	-0.55	
$\lvert s_V - s_A \rvert^3$:condition:phase	0.04 (-0.82, 0.89)	0.08	

S3.2 Visual ventriloquism effects

Table S2. Linear mixed-effects model (LMM) analysis for the visual ventriloquism effects from the pre- and post-learning phases of the main experiment. Test statistics reveal whether the estimated beta is significantly different from 0 (greater absolute t-value indicates that β is further away from 0) and the results of model comparison between the null and alternative models (larger χ^2 indicates stronger evidence for the alternative models).

	Estimated β (Confidence interval)	Test statistics and p-value	
		Significance of β compared to 0 (t-value)	Model comparson

Intercept	0.20 (0.00, 0.41)	2.04	-
$\lvert s_V - s_A \rvert$	0.45 (0.30, 0.60)	5.88	$\chi^2(3) = 102.86$, $p < 0.001***$
$\lvert s_V - s_A \rvert^2$	0.02 (-0.12, 0.15)	0.24	
$\lvert s_V - s_A \rvert^3$	-0.03 (-0.15, 0.09)	-0.47	
Condition	-0.15 (-0.25, -0.06)	-3.12	$\chi^2(1) = 18.83$, $p < 0.001***$
Phase	0.04 (-0.05, 0.14)	0.84	$\chi^2(1) = 1.43$, $p = 0.23$
Condition:phase	-0.01 (-0.15, 0.13)	-0.13	$\chi^2(1) = 0.08$, $p = 0.78$
$\lvert s_V - s_A \rvert$:condition	-0.16 (-0.38, 0.05)	-1.51	$\chi^2(3) = 2.57$, $p = 0.46$
$\lvert s_V - s_A \rvert^2$:condition	-0.02 (-0.21, 0.17)	-0.22	
$\lvert s_V - s_A \rvert^3$:condition	0.03 (-0.14, 0.20)	0.32	
$\lvert s_V - s_A \rvert$:phase	-0.04 (-0.26, 0.17)	-0.40	$\chi^2(3) = 0.07$, $p = 1.00$
$\lvert s_V - s_A \rvert^2$:phase	0.02 (-0.17, 0.21)	0.19	
$\lvert s_V - s_A \rvert^3$:phase	0.06 (-0.10, 0.23)	0.75	
$\lvert s_V - s_A \rvert$:condition:phase	0.1 (-0.21, 0.40)	0.63	$\chi^2(3) = 1.98$, $p = 0.58$
$\lvert s_V - s_A \rvert^2$:condition:phase	-0.07 (-0.34, 0.20)	-0.51	
$\lvert s_V - s_A \rvert^3$:condition:phase	-0.13 (-0.37, 0.11)	-1.05	

S3.3 Unity judgments

$$r_{\text{unity}} \sim 1 + \lvert s_V - s_A \rvert + \text{condition} + \text{phase} + \lvert s_V - s_A \rvert * \text{condition} * \text{phase} + (1 \vert \text{participant})$$

Table S3. Generalized linear mixed-effects model (GLMM) analysis for the binary unity judgments from the pre- and post-learning phases of the main experiment. Test statistics reveal whether the estimated beta is significantly different from 0 (greater absolute z-value indicates that β is further away from 0) and the results of model comparison between the null and alternative models (larger χ^2 indicates stronger evidence for the alternative models).

	Estimated β (Confidence interval)	Test statistics and p-value	
		Significance of β compared to 0 (t-value)	Model comparson
Intercept	0.09 (-0.35, 0.53)	$z = 0.42$, $p = 0.68$	-
$\lvert s_V - s_A \rvert$	-2.90 (-3.08, -2.72)	$z = -31.43$, $p < 0.001$ ***	$\chi^2(3) = 4428.39$, $p < 0.001***$
$\lvert s_V - s_A \rvert^2$	0.17 (0.02, 0.32)	$z = 2.20$, $p = 0.03$ *	
$\lvert s_V - s_A \rvert^3$	0.16 (0.04, 0.28)	$z = 2.57$, $p = 0.01$ *	
Condition	0.14 (0.03, 0.25)	$z = 2.53$, $p = 0.01$ *	$\chi^2(1) = 14.56$, $p < 0.001***$

Phase	-0.03 (-0.14, 0.08)	$z = -0.52$, $p = 0.60$	$\chi^2(1) = 3.37$, $p = 0.07$
Condition:phase	-0.09 (-0.24, 0.07)	$z = -1.13$, $p = 0.26$	$\chi^2(1) = 0.17$, $p = 0.68$
$\lvert s_V - s_A \rvert$:condition	0.07 (-0.18, 0.32)	$z = 0.57$, $p = 0.57$	$\chi^2(3) = 4.13$, $p = 0.25$
$\lvert s_V - s_A \rvert^2$:condition	-0.04 (-0.25, 0.17)	$z = -0.37$, $p = 0.71$	
$\lvert s_V - s_A \rvert^3$:condition	-0.02 (-0.19, 0.14)	$z = -0.29$, $p = 0.77$	
$\lvert s_V - s_A \rvert$:phase	-0.18 (-0.44, 0.08)	$z = -1.38$, $p = 0.17$	$\chi^2(3) = 12.74$, $p < 0.01**$
$\lvert s_V - s_A \rvert^2$:phase	0.08 (-0.14, 0.30)	$z = 0.72$, $p = 0.47$	
$\lvert s_V - s_A \rvert^3$:phase	-0.13 (-0.30, 0.04)	$z = -1.49$, $p = 0.14$	
$\lvert s_V - s_A \rvert$:condition:phase	-0.27 (-0.64, 0.10)	$z = -1.46$, $p = 0.15$	$\chi^2(3) = 7.51$, $p = 0.06$
$\lvert s_V - s_A \rvert^2$:condition:phase	-0.09 (-0.40, 0.22)	$z = -0.59$, $p = 0.55$	
$\lvert s_V - s_A \rvert^3$:condition:phase	0.20 (-0.04, 0.44)	$z = 1.64$, $p = 0.10$	

S4 Individual-level empirical data and model predictions

S4.1 Participants whose common-cause prior about vision and audition was updated in the direction of the new stimulus statistics

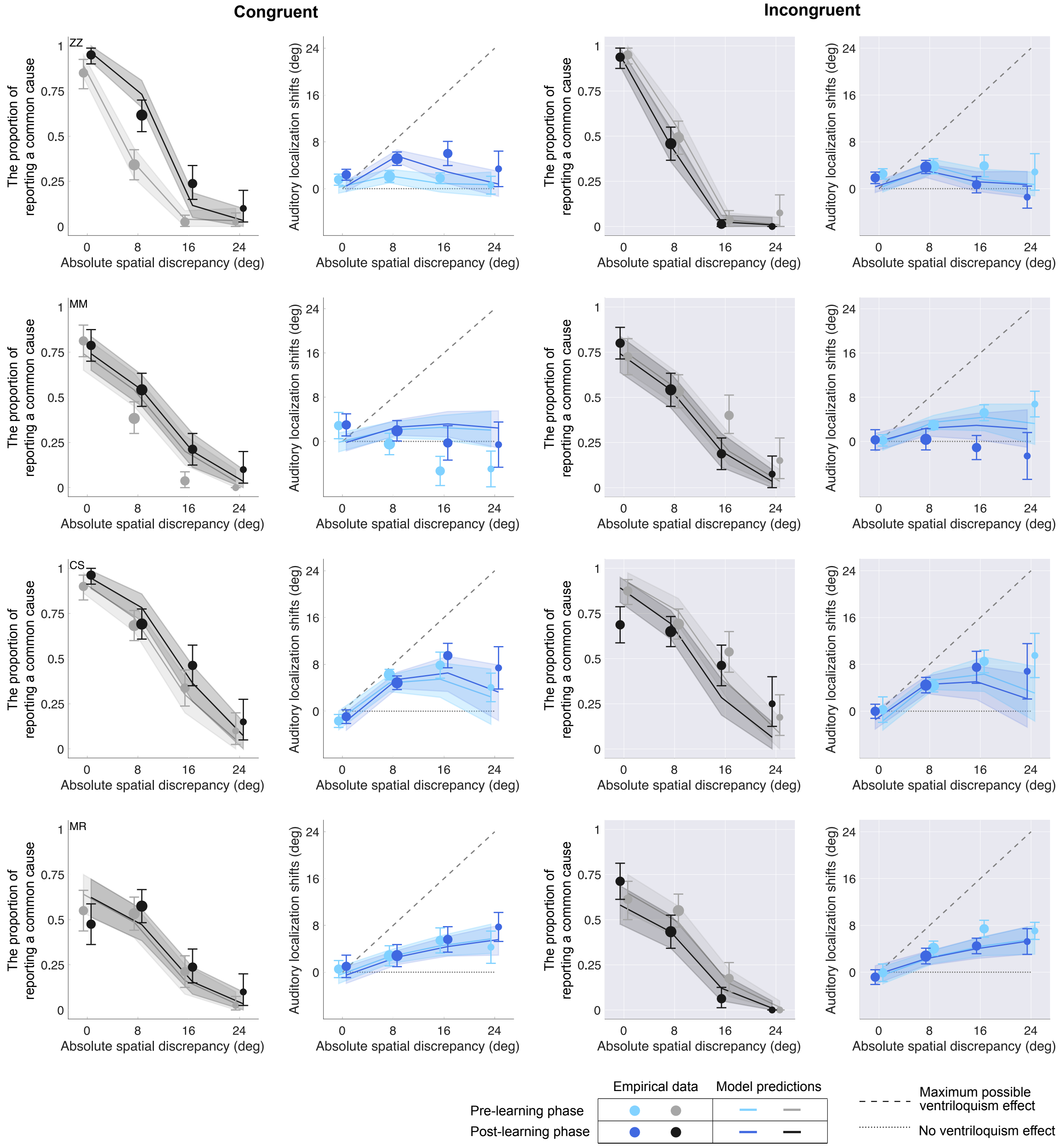

Figure S3. Empirical data (dots) and model predictions (lines) for all the participants from the first group in Figure 5B.

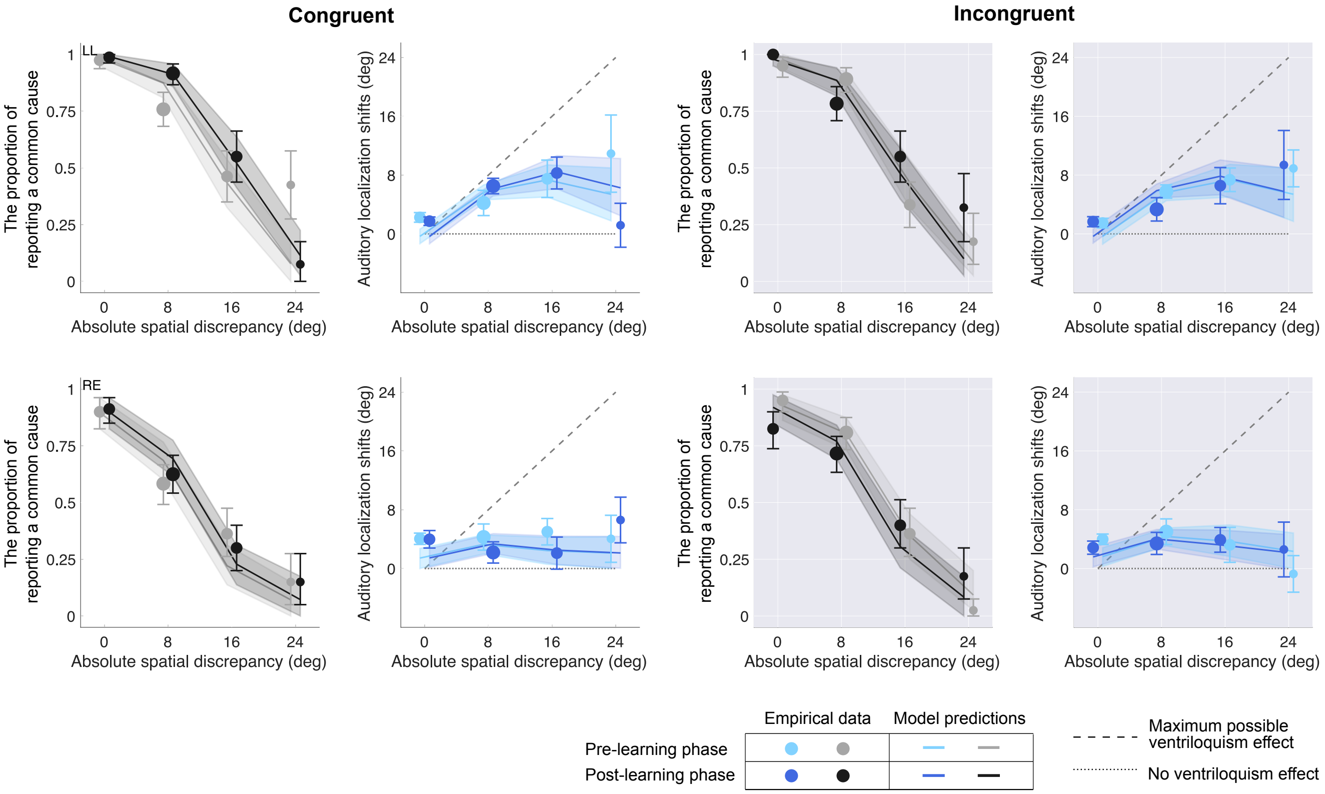

Figure S3. Continued.

S4.2 Participants whose spatial common-cause prior about vision and audition was updated in the opposite direction of stimulus statistics

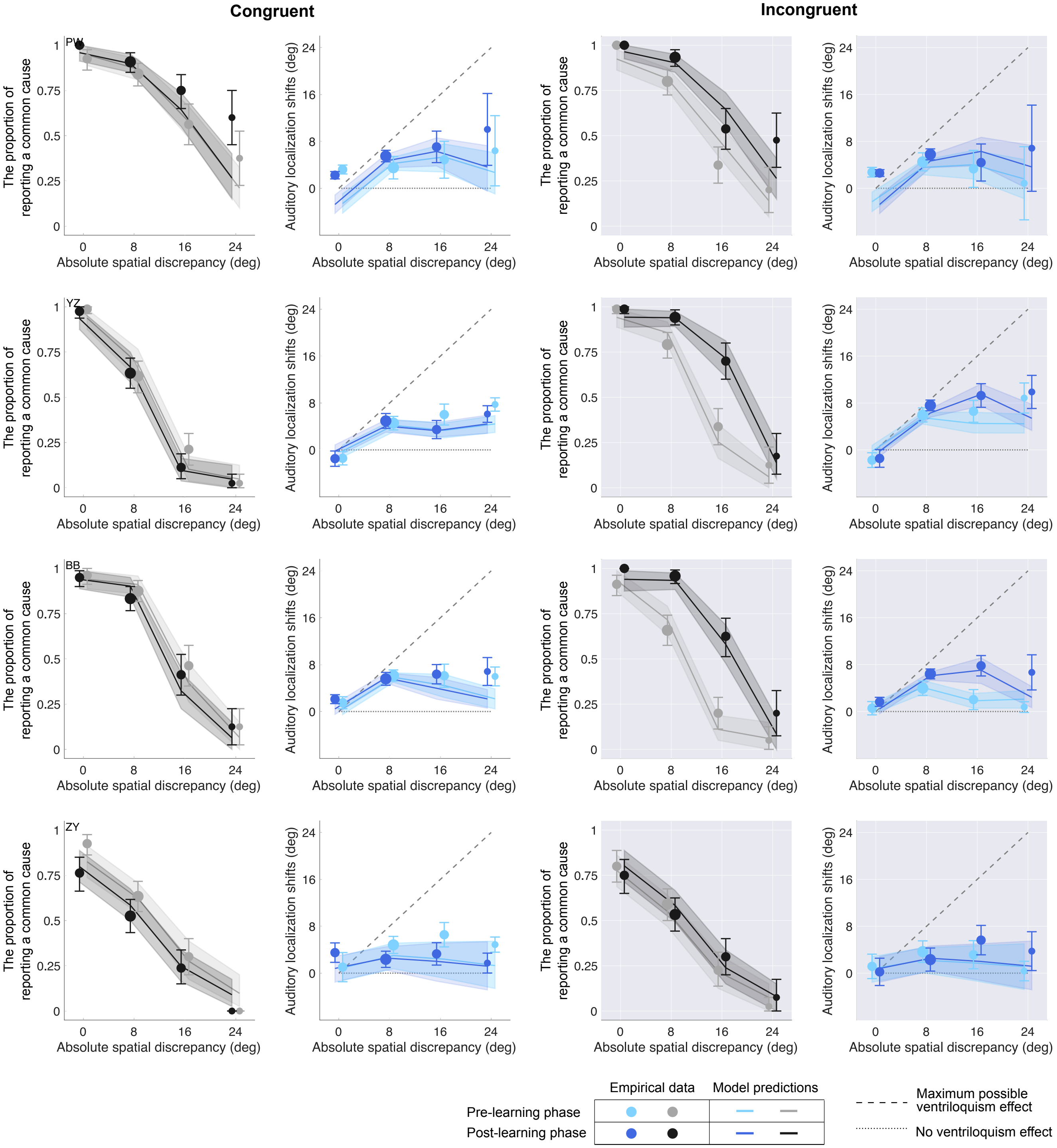

Figure S4. Empirical data (dots) and model predictions (lines) for all the participants from the second group in Figure 5B.

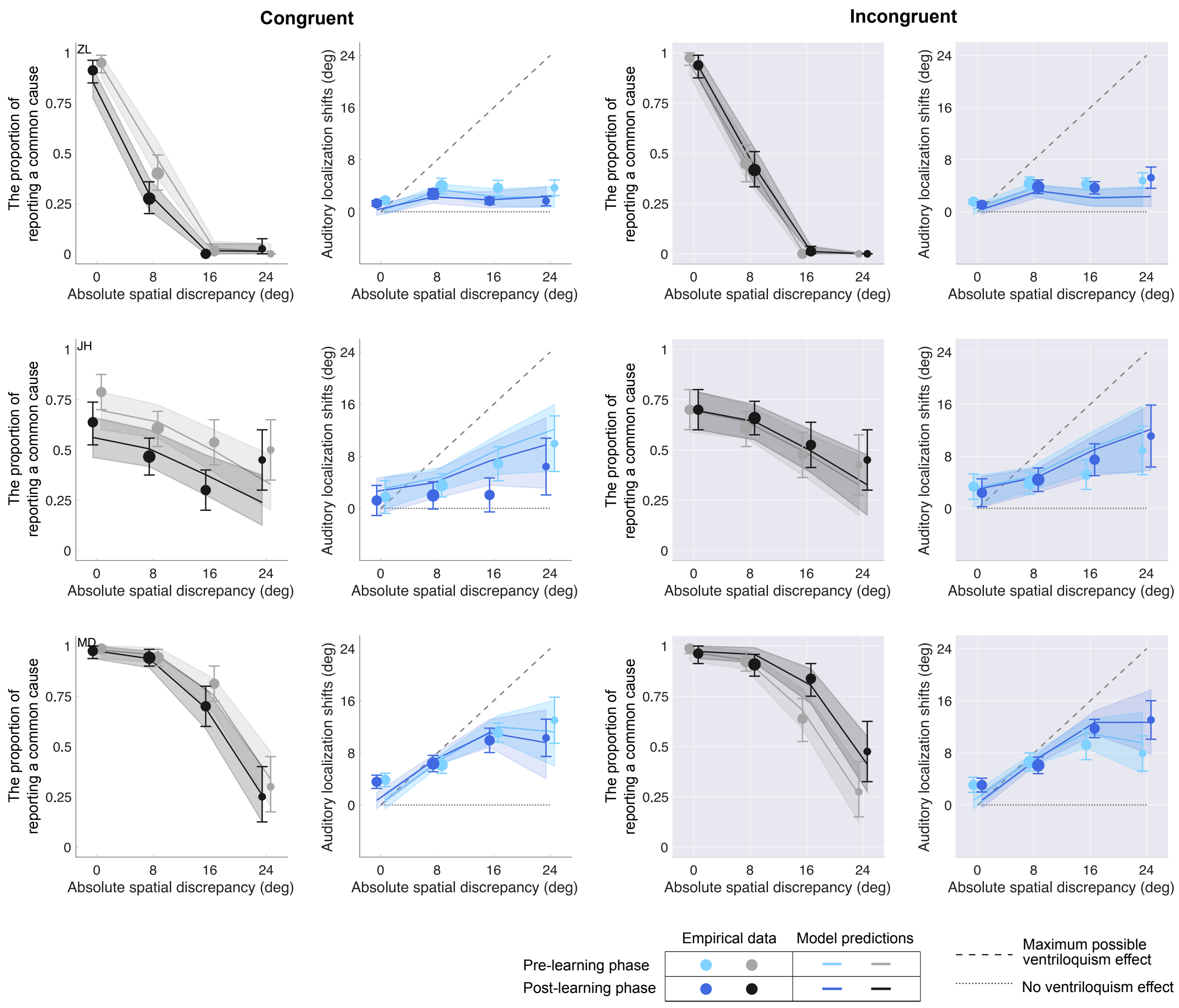

Figure S4. Continued.

S5 Modeling

S5.1 An alternative decision strategy for implicit causal inference - model selection

We considered model selection as an alternative decision strategy for deriving the final auditory and visual location estimates[6]. According to model selection, the final location estimate $\hat{s}'_{AV_l,A}$ is the location estimate conditioned on a common cause $\hat{s}'_{AV_l,A,C=1}$ if the posterior probability of a common cause is greater than that of separate causes. Otherwise, the final location estimate $\hat{s}'_{AV_l,A}$ equals the location estimate conditioned on separate causes $\hat{s}'_{AV_l,A,C=2}$, i.e.,

$$\hat{s}'_{AV_l,A} = \begin{cases} \hat{s}'_{AV_l,A,C=1}, & \text{if } P(C=1|m'_{AV_l,A}, m'_{AV_l,V}) > 0.5 \\ \hat{s}'_{AV_l,A,C=2}, & \text{otherwise.} \end{cases} \tag{S1}$$

Analogously,

$$\hat{s}'_{AV_l,V} = \begin{cases} \hat{s}'_{AV_l,V,C=1}, & \text{if } P(C=1|m'_{AV_l,A}, m'_{AV_l,V}) > 0.5 \\ \hat{s}'_{AV_l,V,C=2}, & \text{otherwise.} \end{cases} \tag{S2}$$

S5.2 Alternative decision strategies for explicit causal inference

S5.2.1 Optimal posterior-based unity judgments

The first alternative decision strategy we considered is that the observer makes a decision about whether a co-occurring auditory and visual stimulus originate from a common source based on the posterior probability of a common cause, $P(C = 1|m'_{AV_l,A}, m'_{AV_l,V})$. More specifically, if this probability is greater than 0.5, then the observer intends to report 'common cause'. In other words,

$$I_{C=1} = \begin{cases} 1, & \text{if } P(C=1|m'_{AV_l,A}, m'_{AV_l,V}) > 0.5 \\ 0, & \text{otherwise.} \end{cases} \tag{S3}$$

$I_{C=1}$ is what the observer intends to report. However, as described in the main text, we assume that the observer lapses occasionally during the task with a lapse rate λ_{unity}. Therefore, even when the observer intends to report 'common cause', he or she will still mistakenly report 'separate causes' with a probability of λ_{unity}, which results in reporting 'common cause' with a probability of $1-\lambda_{\text{unity}}$. In other words,

$$P(I_{C=1} = 1|m'_{AV_l,A}, m'_{AV_l,V}) = \begin{cases} 1-\lambda_{\text{unity}}, & \text{if } P(C=1|m'_{AV_l,A}, m'_{AV_l,V}) > 0.5 \\ \lambda_{\text{unity}}, & \text{otherwise.} \end{cases} \tag{S4}$$

The lapse rate is modeled in the same way for the following alternative decision strategy.

S5.2.2 measurement-based (heuristic) unity judgments

We also considered a heuristic strategy as an alternative, which is based directly on the difference between the auditory and visual measurements. If the difference between them is below ε, then the observer intends to report 'common cause'. That is,

$$P(I_{C=1} = 1|m'_{AV_l,A}, m'_{AV_l,V}) = \begin{cases} 1-\lambda_{\text{unity}}, & \text{if } |m'_{AV_l,A} - m'_{AV_l,V}| < \varepsilon \\ \lambda_{\text{unity}}, & \text{otherwise.} \end{cases} \tag{S5}$$

S5.3 Overview of models with different decision strategies and assumption about the plasticity of the common-cause prior

As described in the main text, we tested three models with different assumptions about the plasticity of the common-cause prior (high-plasticity, short-lasting changes; high-plasticity, long-lasting changes; and no-plasticity). Each of these three models was tested five times with different assumptions about the decision strategy for deriving the final location estimates as well as determining the unity judgments. Specifically, we considered model averaging paired with estimate-based (heuristic), posterior-based, or measurement-based (heuristic) unity judgments. We also considered model selection paired with either posterior-based, or measurement-based (heuristic), but not estimate-based (heuristic) unity judgments. This is because having both assumptions makes the model logically circular: the estimates are based on the unity judgment, which, in turn, is based on the estimates. Therefore, we tested a total of five decision strategies for each of the three models for the common-cause prior, resulting in a total of 15 models (Table S5.3).

Table S4. Summary of the total number of free parameters for all the tested models. Among models with the same assumption for the plasticity of the common-cause prior, those assuming estimate-based and measurement-based unity judgments have one extra free parameter (ε) than those assuming posterior-based unity judgments.

Decision strategy		Model		
Implicit causal inference	Explicit causal inference	High-plasticity, short-lasting changes	High-plasticity, long-lasting changes	No-plasticity
Model averaging	Estimate-based (heuristic)	14	15	12
	Posterior-based	13	14	11
	Measurement-based (heuristic)	14	15	12
Model selection	Posterior-based	13	14	11
	Measurement-based (heuristic)	14	15	12

S5.4 Model likelihoods

For a given model M, we fitted the model jointly to the forced-choice data from the bimodal spatial-discrimination task X_1, the localization responses from the unimodal spatial-localization task X_2, and the localization responses and unity judgments from the bimodal spatial-localization task during the pre- and post-learning phases of both congruent and incongruent conditions X_3. The localization responses from the localization practice task were fitted separately to reduce the number of free parameters estimated simultaneously. The data from the learning phase were excluded as they are sequentially dependent, making them computationally challenging to fit. All models were fit using a maximum-likelihood procedure. That is, a set of free parameters Θ was chosen to maximize the log likelihood

$$\log P(X|M,\Theta) = \sum_{k=1}^{3} \log P(X_k|M,\Theta_k), \tag{S6}$$

where $X_1, X_2, X_3 \subset X$ and $\Theta_1, \Theta_2, \Theta_3 \subset \Theta$. The candidate models only varied with respect to Θ_3.

S5.4.1 Model likelihoods given the bimodal spatial-discrimination task

For each trial t, participants were presented with an auditory test stimulus at location $s_{A,o(t)}$, and a visual standard stimulus at location $s_{V,w(t)}$, in a random order. Participants were then asked to indicate whether the auditory stimulus was located to the left, $I_{\text{A-right},w(t),o(t)} = 0$, or to the right, $I_{\text{A-right},w(t),o(t)} = 1$, compared to the location of the visual stimulus. For each such trial, the likelihood of a model M and a candidate parameter set Θ_1 given the response $I_{\text{A-right},w(t),o(t)}$ is

$$P(I_{\text{A-right},w(t),o(t)} = 1|M,\Theta_1) = p_{I_{\text{A-right},w(t),o(t)}=1}{}^{I_{\text{A-right},w(t),o(t)}=1} \cdot (1 - p_{I_{\text{A-right},w(t),o(t)}=1})^{1-I_{\text{A-right},w(t),o(t)}=1}, \tag{S7}$$

where $p_{I_{\text{A-right},w(t),o(t)}=1}$ is defined in Eq. 15. Thus, the log likelihood given the responses across all T_1 trials is

$$\log P(X_1|M,\Theta_1) = \sum_{t=1}^{T_1} \left[I_{\text{A-right},w(t),o(t)} = 1 \cdot \log p_{I_{\text{A-right},w(t),o(t)}=1} + \left(1 - I_{\text{A-right},w(t),o(t)} = 1\right) \cdot \log\left(1 - p_{I_{\text{A-right},w(t),o(t)}=1}\right) \right]. \tag{S8}$$

$p_{I_{\text{A-right},w(t),o(t)}=1}$ depends on the bias parameters a_A and b_A, the parameters of the supra-modal prior over locations μ'_P and σ'_P, the measurement noise σ'_A and σ'_V, as well as the lapse rate λ_{AV}. The final set of free parameters that were constrained by the forced-choice data in this task was $\Theta_1 = \{a_A, b_A, \sigma'_A, \sigma'_V, \sigma'_P, \lambda_{AV}\}$.

S5.4.2 Model likelihoods given unimodal localization responses

For each trial t, participants were asked to localize either a unimodal visual or auditory stimulus, resulting in a cursor location setting $r_{A,u(t)}$ or $r_{V,w(t)}$. The localization responses from this task were modeled as Gaussian distributions. From these distributions, we can compute the likelihood of a model M and a candidate parameter set Θ_2 as the Gaussian probability density function in Eq. 13, evaluated at the observed localization response $r_{A,u(t)}$ or $r_{V,w(t)}$:

$$P(r_{A,u(t)}|M,\Theta_2) = \frac{1}{\sqrt{2\pi(\sigma^2_{\hat{s}'_A} + \sigma^2_r)}} e^{-\frac{\left(r_{A,u(t)} - \mu'_{\hat{s}_{A,u(t)}}\right)^2}{2\left(\sigma^2_{\hat{s}'_A} + \sigma^2_r\right)}}, \text{ and } P(r_{V,w(t)}|M,\Theta_2) = \frac{1}{\sqrt{2\pi(\sigma^2_{\hat{s}'_V} + \sigma^2_r)}} e^{-\frac{\left(r_{V,w(t)} - \mu'_{\hat{s}_{V,w(t)}}\right)^2}{2\left(\sigma^2_{\hat{s}'_V} + \sigma^2_r\right)}}. \tag{S9}$$

The log-likelihood is the sum of the log-likelihoods across trials:

$$\log P(X_2|M,\Theta_2) = \sum_{t_A} \log P(r_{A,u(t)}|M,\Theta_2) + \sum_{t_V} \log P(r_{V,w(t)}|M,\Theta_2). \tag{S10}$$

The log-likelihood depends on $\mu_{\hat{s}_{A,u(t_A)}}$ and $\mu_{\hat{s}_{A,u(t_A)}}$, which in turn depend on the bias parameters a_A and b_A, the parameters of the supra-modal prior over locations μ'_P and σ'_P, the standard deviation of measurement distributions σ'_A and σ'_V, as well as the perception-unrelated response noise σ_r, which was estimated from the data from the localization-practice task. Therefore, the set of parameters constrained by the localization responses in this task is $\Theta_2 = \{a_A, b_A, \sigma'_A, \sigma'_V, \sigma'_P\}$.

S5.4.3 Model likelihoods given bimodal spatial-localization responses and unity judgments

The main experiment consisted of two conditions ($i = 1$: congruent; $i = 2$: incongruent) and two phases ($j = 1$: pre-learning; $j = 2$: post-learning). For each trial t of session (i, j), participants were presented with an audiovisual stimulus pair AV_l (l indexes audiovisual pair, $l \in \{1,2,...,16\}$). After stimulus presentation, participants first localized a stimulus of a cued modality, which resulted in a cursor location setting $r_{i,j,AV_{l(t)},A}$ or $r_{i,j,AV_{l(t)},V}$. Participants then made a unity judgment, which resulted in a binary response $I_{C=1,i,j,AV_{l(t)}}$ (1: same source; 0: different sources). The overall log-likelihood of M and Θ_M (where we designate the version of Θ_3 used for model M as Θ_M) is the log-likelihood summed over all localization responses and unity judgments:

$$\begin{aligned}
&\log P(X|M,\Theta_M) = \sum_{i=1}^{2}\sum_{j=1}^{2}\sum_{l=1}^{16}\sum_{t=1}^{10} \\
&\log\left(\iint P(r_{i,j,AV_{l(t)},A}, I_{C=1,i,j,AV_{l(t)}}|m'_{AV_l,A}, m'_{AV_l,V})P(m'_{AV_l,A}, m'_{AV_l,V}|s_{i,j,AV_l})dm'_{AV_l,A}dm'_{AV_l,V}\right) \\
&+\log\left(\iint P(r_{i,j,AV_{l(t)},V}, I_{C=1,i,j,AV_{l(t)}}|m'_{AV_l,A}, m'_{AV_l,V})P(m'_{AV_l,A}, m'_{AV_l,V}|s_{i,j,AV_l})dm'_{AV_l,A}dm'_{AV_l,V}\right) \\
&= \log\left(\int\int P(r_{i,j,AV_{l(t)},A}|m'_{AV_l,A}, m'_{AV_l,V})P(I_{C=1,i,j,AV_{l(t)}}|m'_{AV_l,A}, m'_{AV_l,V})\right. \\
&\left. P(m'_{AV_l,A}|s_{i,j,AV_l})P(m'_{AV_l,V}|s_{i,j,AV_l})dm'_{AV_l,A}dm'_{AV_l,V}\right) \\
&+\log\left(\int\int P(r_{i,j,AV_{l(t)},V}|m'_{AV_l,A}, m'_{AV_l,V})P(I_{C=1,i,j,AV_{l(t)}}|m'_{AV_l,A}, m'_{AV_l,V})\right. \\
&\left. P(m'_{AV_l,A}|s_{i,j,AV_l})P(m'_{AV_l,V}|s_{i,j,AV_l})dm'_{AV_l,A}dm'_{AV_l,V}\right).
\end{aligned} \tag{S11}$$

The log-likelihood depends on the final location estimates $\hat{s}_{AV_l,A}$ and $\hat{s}_{AV_l,V}$, which in turn depend on the bias parameters a_A and b_A, the parameters of the supra-modal prior over locations μ'_P and σ'_P, the standard deviation of measurement distributions $\sigma'_{AV,A}$ and $\sigma'_{AV,V}$ under bimodal presentation mode, as well as two lapse rates λ_{unity} (one for each condition), the set of common-cause priors $\{p_{c=1}\}$ that are model-specific, and the perception-unrelated response noise σ_r, which was estimated using the data from the localization-practice task. Additionally, the log-likelihood depends on ε, the internal criterion for determining the unity judgment. Therefore, the set of parameters constrained by the localization responses in this task is $\Theta_3 = \{a_A, b_A, \sigma'_{AV,A}, \sigma'_{AV,V}, \sigma'_P, \varepsilon, \{p_{c=1}\}, \{\lambda_{\text{unity, cong}}, \lambda_{\text{unity, incong}}\}\}$.

S5.4.4 Model likelihoods of models that assume different decision strategies

As described in the previous section, the likelihood of models that assume model averaging as the decision strategy for deriving final location estimates is the joint likelihood of a pair of auditory and visual sensory measurements given the localization response registered by the cursor and the unity judgment $P(r_{AV_l}, I_{C=1,AV_l}|m'_{AV_l,A}, m'_{AV_l,V})$, multiplied by the joint probability of the two measurements $P(m'_{AV_l,A}, m'_{AV_l,V}|s_{AV_l})$, integrated over the two variables inaccessible to the experimenter - the visual and auditory sensory measurements. Since the models assume model averaging, the localization response r_{AV_l} and the unity judgment $I_{C=1,AV_l}$ are conditionally independent regardless of which decision strategy the unity judgment is based on. Thus, $P(r_{AV_l}, I_{C=1,AV_l}|m'_{AV_l,A}, m'_{AV_l,V})$ can be rewritten as the product of the likelihood of a pair of auditory and visual sensory measurements given the localization response $P(r_{AV_l}|m'_{AV_l,A}, m'_{AV_l,V})$, and the likelihood given the unity judgment $P(I_{C=1,AV_l}|m'_{AV_l,A}, m'_{AV_l,V})$. However, this conditional independence does not apply to models that assume model selection.

Based on model selection, the final location estimate depends on the unity judgment. Therefore, Eq. S11 is modified as

$$\begin{aligned}
\log P(X|M,\Theta_M) &= \sum_{i=1}^{2}\sum_{j=1}^{2}\sum_{l=1}^{16}\sum_{t=1}^{10} \\
&= \log\Big(\int\int P(r_{i,j,AV_{l(t)},A}|I_{C=1,i,j,AV_{l(t)}}, m'_{AV_l,A}, m'_{AV_l,V})P(I_{C=1,i,j,AV_{l(t)}}|m'_{AV_l,A}, m'_{AV_l,V}) \\
&\qquad P(m'_{AV_l,A}|s_{i,j,AV_l})P(m'_{AV_l,V}|s_{i,j,AV_l})dm'_{AV_l,A}dm'_{AV_l,V}\Big) \\
&+ \log\Big(\int\int P(r_{i,j,AV_{l(t)},V}|I_{C=1,i,j,AV_{l(t)}}, m'_{AV_l,A}, m'_{AV_l,V})P(I_{C=1,i,j,AV_{l(t)}}|m'_{AV_l,A}, m'_{AV_l,V}) \\
&\qquad P(m'_{AV_l,A}|s_{i,j,AV_l})P(m'_{AV_l,V}|s_{i,j,AV_l})dm'_{AV_l,A}dm'_{AV_l,V}\Big),
\end{aligned} \tag{S12}$$

where

$$P(r_{i,j,AV_{l(t)},A}|I_{C=1,i,j,AV_{l(t)}}, m'_{AV_l,A}, m'_{AV_l,V}) = \begin{cases} \phi(r_{i,j,AV_{l(t)},A}; \hat{s}'_{i,j,AV_{l(t)},A,C=1}, \sigma_r^2), \text{ if } I_{C=1,i,j,AV_{l(t)}} = 1 \\ \phi(r_{i,j,AV_{l(t)},A}; \hat{s}'_{i,j,AV_{l(t)},A,C=2}, \sigma_r^2), \text{ if } I_{C=1,i,j,AV_{l(t)}} = 0, \end{cases} \tag{S13}$$

and analogously,

$$P(r_{i,j,AV_{l(t)},V}|I_{C=1,i,j,AV_{l(t)}}, m'_{AV_l,A}, m'_{AV_l,V}) = \begin{cases} \phi(r_{i,j,AV_{l(t)},V}; \hat{s}'_{i,j,AV_{l(t)},V,C=1}, \sigma_r^2), \text{ if } I_{C=1,i,j,AV_{l(t)}} = 1 \\ \phi(r_{i,j,AV_{l(t)},V}; \hat{s}'_{i,j,AV_{l(t)},V,C=2}, \sigma_r^2), \text{ if } I_{C=1,i,j,AV_{l(t)}} = 0. \end{cases} \tag{S14}$$

S6 Mathematical derivations

S6.1 The center and the spread of the distribution for localization responses

A presentation of an auditory stimulus at location $s_{A,u}$, where u indexes participant-specific auditory stimulus location, results in an internal measurement $m'_{A,u}$ with variability of ${\sigma'_A}^2$. The estimate of the remapped location of this stimulus is the average of the internal measurement and the mean of the spatial prior μ'_P, each weighted by their relative reliabilities, respectively:

$$\hat{s}'_{A,u} = \frac{m'_{A,u}{\sigma'_A}^{-2} + \mu'_P {\sigma'_P}^{-2}}{{\sigma'_A}^{-2} + {\sigma'_P}^{-2}} = c_A m'_{A,u} + f_A, \text{ where } c_A = \frac{{\sigma'_A}^{-2}}{{\sigma'_A}^{-2} + {\sigma'_P}^{-2}} \text{ and } f_A = \frac{\mu'_P {\sigma'_P}^{-2}}{{\sigma'_A}^{-2} + {\sigma'_P}^{-2}}. \tag{S15}$$

Analogously, the estimate of the remapped location of a visual stimulus is:

$$\hat{s}'_{V,w} = \frac{m'_{V,w}{\sigma'_V}^{-2} + \mu'_P {\sigma'_P}^{-2}}{{\sigma'_V}^{-2} + {\sigma'_P}^{-2}} = c_A m'_{V,w} + f_A, \text{ where } c_V = \frac{{\sigma'_V}^{-2}}{{\sigma'_V}^{-2} + {\sigma'_P}^{-2}} \text{ and } f_V = \frac{\mu'_P {\sigma'_P}^{-2}}{{\sigma'_V}^{-2} + {\sigma'_P}^{-2}}, \tag{S16}$$

where w indexes the visual stimulus location ($s_V \in \{-12, -4, 4, 12°\}$). Given that the measurement distributions are Gaussian and the family of Gaussian distributions is closed under linear transformations, the probability distribution of the location estimates of a test stimulus is

$$\hat{s}'_{A,u} \sim \mathcal{N}(\mu_{\hat{s}_{A,u}}, \sigma^2_{\hat{s}'_A}) = \mathcal{N}(c_A s'_{A,u} + f_A, c_A^2 {\sigma'_A}^2) = \mathcal{N}(c_A(a_A s_{A,u} + b_A) + f_A, c_A^2 {\sigma'_A}^2), \tag{S17}$$

where

$$\mu_{\hat{s}'_{A,u}} = c_A(a_A s_{A,u} + b_A) + f_A = \frac{(a_A s_{A,u} + b_A){\sigma'_A}^{-2} + \mu'_P {\sigma'_P}^{-2}}{{\sigma'_A}^{-2} + {\sigma'_P}^{-2}} \text{ and } \sigma^2_{\hat{s}'_A} = \left(\frac{{\sigma'_A}^{-2}}{{\sigma'_A}^{-2} + {\sigma'_P}^{-2}}\right)^2 {\sigma'_A}^2. \tag{S18}$$

Analogously, for the location estimates of the visual standard stimulus,

$$\hat{s}'_{V,w} \sim \mathcal{N}(c_V s_{V,w} + f_V, c_V^2 {\sigma'_V}^2), \tag{S19}$$

where

$$\mu_{\hat{s}'_{V,w}} = c_V s_{V,w} + f_V = \frac{s_{V,w}{\sigma'_V}^{-2} + \mu'_P {\sigma'_P}^{-2}}{{\sigma'_V}^{-2} + {\sigma'_P}^{-2}}, \text{ and } \sigma^2_{\hat{s}'_V} = \left(\frac{{\sigma'_V}^{-2}}{{\sigma'_V}^{-2} + {\sigma'_P}^{-2}}\right)^2 {\sigma'_V}^2. \tag{S20}$$

S6.2 The psychometric functions for the bimodal spatial-discrimination task

In this task, visual stimuli were presented at four different locations $s_{V,w}$, where w indexes the visual stimulus location ($s_V \in \{-12, -4, 4, 12°\}$). In each trial, $s_{V,w}$ was paired with an auditory stimulus, at one of test locations $s_{A,o}$, where o indexes the finer grid of auditory locations. For each pair, the model predicts $p_{w,o}$, the probability of estimating the auditory test stimulus $s_{A,o}$ to be located to the right of the visual standard stimulus at location $s_{V,w}$.

We assume that the observer makes the decision by comparing the internal location estimates $\hat{s}'_{A,o}$ and $\hat{s}'_{V,w}$,

$$p_{w,o} = P(\hat{s}'_{A,o} > \hat{s}'_{V,w}) = P(\hat{s}'_{A,o} - \hat{s}'_{V,w} > 0). \tag{S21}$$

Given Eqs. S15-S16, the probability distribution of the difference between the two location estimates $\hat{s}'_{A,o}$ and $\hat{s}'_{V,w}$ can be written as

$$\hat{s}'_{A,o} - \hat{s}'_{V,w} \sim \mathcal{N}(\mu_{\hat{s}'_{A,o}} - \mu_{\hat{s}'_{V,w}}, {\sigma_{\hat{s}'_A}}^2 + {\sigma_{\hat{s}'_V}}^2). \tag{S22}$$

Taken together, the probability of perceiving an auditory test stimulus at presented location $s_{A,o}$ to the right of a visual standard stimulus presented at location $s_{V,w}$ is

$$\begin{aligned} p_{w,o} &= P(\hat{s}'_{A,o} > \hat{s}'_{V,w}) = P(\hat{s}'_{A,o} - \hat{s}'_{V,w} > 0) = 1 - \Phi(0; \mu_{\hat{s}'_{A,o}} - \mu_{\hat{s}'_{V,w}}, {\sigma_{\hat{s}'_A}}^2 + {\sigma_{\hat{s}'_V}}^2) \\ &= 1 - \Phi(0; c_A(a_A s_{A,o} + b_A) + f_A - (c_V s_{V,w} + f_V), c_A^2 {\sigma'_A}^2 + c_V^2 {\sigma'_V}^2). \end{aligned} \tag{S23}$$

We additionally assumed that participants lapsed occasionally at rate λ_{AV}. Therefore, the probability of reporting an auditory test stimulus as being farther to the right of a visual test stimulus is equal to

$$p_{r_{w,o}=1} = 0.5\lambda_{AV} + (1 - \lambda_{AV}) p_{w,o}. \tag{S24}$$

In sum, the probability of reporting the auditory stimulus as to the right of the visual stimulus as a function of the distance between the two stimulus locations is described by a cumulative Gaussian distribution that approaches $\lambda_{AV}/2$ when the auditory stimulus is located far to the left and $1 - \lambda_{AV}/2$ when it is far to the right of the visual stimulus.

S7 Estimated parameters given the best-fitting model

S7.1 All relevant free model parameters

Table S5. Estimated parameters given the best-fitting model for each participants. Note that the elements in $\overrightarrow{p_{C=1}}$ follows the order of pre-learning, congruent post-learning, and incongruent post-learning if the best-fitting model is the high-plasticity, short-lasting changes model. $\overrightarrow{p_{C=1}}$ follows the order of congruent pre-learning, congruent post-learning, incongruent pre-learning, incongruent post-learning if the best-fitting model is the high-plasticity, long-lasting changes model. The elements in $\overrightarrow{\lambda_{\text{unity}}}$ follows this order: congruent and incongruent.

Participant	Estimated parameters given the best-fitting model											
	a_A	b_A	σ'_A	σ'_V	λ_{AV}	σ_r	$\sigma'_{AV,A}$	$\sigma'_{AV,V}$	σ'_P	$\overrightarrow{p_{C=1}}$	$\overrightarrow{\lambda_{\text{unity}}}$	ε
PW	0.78	3.16	8.75	0.93	0.06	1.10	4.44	1.25	30.00	[0.97, 0.99; 0.92, 0.99]	[0.02, 0.02]	4.18
SW	2.36	-2.39	5.49	0.04	0.00	1.78	4.95	0.99	18.38	[0.46]	[0.01, 0.00]	7.88
HL	3.26	-1.12	8.67	2.18	0.06	1.58	6.63	1.47	7.37	[0.68]	[0.03, 0.06]	-
YZ	1.91	-5.14	3.42	1.42	0.06	2.06	5.62	1.95	4.49	[0.67, 0.66; 0.86, 0.99]	[0.05, 0.06]	-
NH	2.30	-10.54	7.22	0.15	0.06	1.64	10.24	2.00	9.91	[0.82]	[0.06, 0.04]	3.25
ZZ	2.35	-3.96	5.02	1.33	0.06	1.54	5.49	0.001	8.00	[0.47, 0.92; 0.75, 0.64]	[0.03, 0.01]	3.38
BB	2.40	-8.70	4.44	2.40	0.06	2.03	5.51	1.62	4.53	[0.96, 0.95; 0.75, 0.99]	[0.06, 0.06]	-
ZY	3.17	-24.68	10.78	1.02	0.06	1.17	11.69	1.42	15.36	[0.54, 0.51; 0.49, 0.51]	[0.06, 0.05]	-
MR	3.66	-8.80	6.60	2.35	0.06	1.90	11.80	2.85	8.52	[0.29, 0.26, 0.20]	[0.02, 0.00]	3.74
ZL	1.55	-3.49	3.14	0.56	0.06	0.83	4.70	1.06	6.22	[0.68, 0.42, 0.64]	[0.01, 0.00]	2.58
RE	2.93	-3.13	5.10	2.18	0.06	2.52	6.59	2.23	6.49	[0.66, 0.67; 0.80, 0.76]	[0.06, 0.06]	-
MM	1.42	-0.70	5.91	0.91	0.00	1.14	7.92	1.97	13.32	[0.37, 0.48; 0.65, 0.44]	[0.00, 0.00]	9.20
LL	1.53	-3.36	4.58	0.003	0.00	1.42	7.67	1.06	10.46	[0.87, 0.91, 0.89]	[0.00, 0.01]	4.88
CS	2.64	-6.63	10.80	1.12	0.00	1.70	9.97	1.61	14.48	[0.81, 0.86; 0.86, 0.78]	[0.01, 0.03]	4.08
JH	3.38	-3.38	10.57	0.80	0.06	1.84	17.30	0.001	29.86	[0.67, 0.50, 0.67]	[0.05, 0.04]	4.02
MD	2.67	-10.62	9.51	1.52	0.06	1.79	9.70	0.001	12.01	[0.98, 0.97; 0.97, 0.99]	[0.02, 0.03]	3.15
HHL	4.00	-1.65	8.98	0.46	0.06	1.00	25.00	0.78	12.97	[0.95]	[0.06, 0.06]	1.06
Mean:	2.49	-5.60	7.00	1.14	0.05	1.59	9.13	1.31	12.49	-	[0.03, 0.03]	-
SEM:	0.21	1.49	0.63	0.19	0.01	0.39	1.28	0.20	1.85	-	[0.01, 0.01]	-

S7.2 Best-fitting models

Table S6. The best-fitting model for all the participants.

Decision strategy		Model		
Implicit causal inference	Explicit causal inference	High-plasticity, short-lasting changes	High-plasticity, long-lasting changes	No-plasticity
Model averaging	Estimate-based (heuristic)	MR, ZL, LL, JH	PW, ZZ, CS, MD	NH, HHL
	Posterior-based	-	-	-

	Measurement-based (heuristic)	-	MM	SW
Model selection	Posterior-based	-	YZ, BB, ZY, RE	HL
	Measurement-based (heuristic)	-	-	-

S7.3 Estimated common-cause priors given the best-fitting model

Table S7. Estimated common-cause priors, changes in the prior, and negative log likelihood of model parameters for participants whose data were best explained by the high-plasticity, short-lasting changes model. Brackets include 95% bootstrapped confidence intervals.

Participant	Estimated common-cause priors $\overrightarrow{p_{C=1}}$			Changes of the common-cause prior		nLL
	Pre-learning	Post-learning				
		Congruent	Incongruent	Congruent	Incongruent	
MR	0.29 (0.20, 0.36)	0.26 (0.11, 0.36)	0.19 (0.10, 0.27)	-0.03 (-0.15, 0.09)	-0.10 (-0.18, 0.02)	4857.2 (4772.7, 4917.0)
ZL	0.68 (0.57, 0.80)	0.41 (0.32, 0.57)	0.64 (0.51, 0.78)	-0.27 (-0.39, -0.13)	-0.04 (-0.18, 0.07)	3658.3 (3557.8, 3771.8)
LL	0.87 (0.82, 0.90)	0.91 (0.88, 0.94)	0.89 (0.84, 0.92)	0.04 (0.01, 0.08)	0.02 (-0.02, 0.07)	3977.4 (3909.8, 4034.5)
JH	0.67 (0.56, 0.70)	0.50 (0.39, 0.56)	0.67 (0.51, 70)	-0.17 (-0.23, -0.10)	0 (-0.11, 0.09)	4905.8 (4784.4, 5026.6)

Table S8. Estimated common-cause priors, changes in the prior, and negative log likelihood of model parameters for participants whose data were best explained by the high-plasticity, long-lasting changes model.

Participant	Estimated common-cause priors $\overrightarrow{p_{C=1}}$				Changes of the common-cause prior		nLL
	Pre-learning		Post-learning				
	Congruent	Incongruent	Congruent	Incongruent	Congruent	Incongruent	
PW	0.98 (0.94, 0.99)	0.92 (0.88, 0.95)	0.99 (0.99, 0.99)	0.99 (0.98, 0.99)	0.01 (0.00, 0.04)	0.07 (0.03, 0.11)	3903.5 (3772.5, 4021.7)
YZ	0.67 (0.60, 0.73)	0.86 (0.76, 0.91)	0.66 (0.59, 0.70)	0.99 (0.97, 0.99)	-0.01 (-0.09, 0.06)	0.13 (0.08, 0.21)	4193.1 (4084.7, 4320.7)
ZZ	0.47 (0.30, 0.56)	0.75 (0.64, 0.85)	0.92 (0.85, 0.96)	0.64 (0.54, 0.75)	0.46 (0.30, 0.60)	-0.11 (-0.23, 0.00)	3938 (3813.8, 4073.9)
BB	0.96 (0.89, 0.98)	0.75 (0.67, 0.81)	0.95 (0.85, 0.98)	0.99 (0.97, 0.99)	-0.01 (-0.1, 0.03)	0.24 (0.18, 0.30)	4152.2 (4000.0, 4261.3)
ZY	0.54 (0.49, 0.57)	0.49 (0.45, 0.51)	0.51 (0.46, 0.54)	0.51 (0.46, 0.53)	-0.03 (-0.07, 0.01)	0.02 (-0.03, 0.07)	4645.9 (4491.0, 4818.4)
RE	0.66 (0.59, 0.74)	0.8 (0.71, 0.88)	0.67 (0.62, 0.74)	0.76 (0.70, 0.83)	0.01 (-0.08, 0.10)	-0.04 (-0.12, 0.04)	4639.6 (4555.3, 4704.4)
MM	0.37 (0.28, 0.44)	0.65 (0.58, 0.70)	0.48 (0.40, 0.55)	0.44 (0.36, 0.51)	0.11 (0.02, 0.18)	-0.21 (-0.29, -0.12)	4582 (4513.8, 4635.6)
CS	0.81 (0.75, 0.85)	0.86 (0.80, 0.90)	0.86 (0.82, 0.92)	0.78 (0.69, 0.88)	0.05 (-0.01, 0.12)	-0.08 (-0.16, 0.02)	4720.5 (4599.7, 4849.2)
MD	0.98 (0.87, 0.99)	0.97 (0.87, 0.98)	0.97 (0.85, 0.98)	0.99 (0.92, 0.99)	-0.01 (-0.03. 0.04)	0.02 (0.00,0.08)	3961.9 (3846.3, 4064.4)

Table S9. Estimated common-cause priors and negative log likelihood of model parameters for participants whose data were best explained by the no-plasticity model.

Participant	Estimated common-cause priors $\overrightarrow{p_{C=1}}$	nLL
SW	0.46 (0.35, 0.54)	4351.1 (4285.0, 4400.4)

HL	0.68<br>(0.63, 0.76)	4185.3<br>(4104.2, 4281.5)
NH	0.82<br>(0.77, 0.87)	4599.5<br>(4489.2, 4678.1)
HHL	0.95<br>(0.94, 0.96)	3507.2<br>(3408.5, 3588.5)

S8 Complete model comparison results

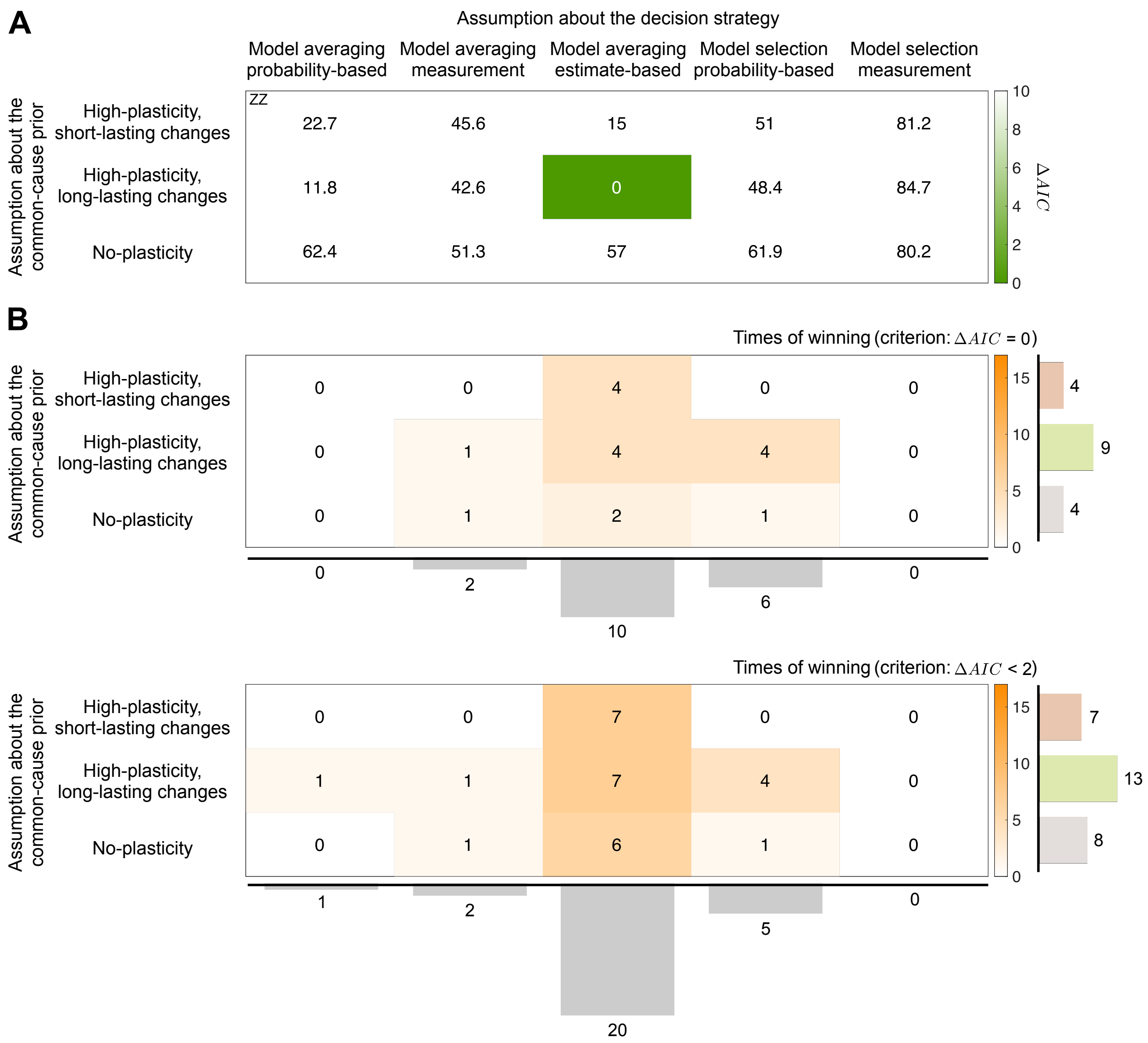

Figure S5. Model comparison. (A) Model performance of all 15 candidate models (column: decision strategies; row: the plasticity of the common-cause prior and the duration of the changes) for a representative participant ZZ. Color key: Δ_{AIC} (more saturated green represents better model performance). Color axis is clipped at 10 for the purpose of visualization. (B) Results of model comparison for all participants (outliers were excluded). Color key: the number of times each model wins (top panel: a winning model has a value of $\Delta_{AIC} = 0$; bottom panel: a winning model has a value of $\Delta_{AIC} < 2$, each relative to the best model for that participant). Color axis is clipped at the total number of participants. Vertical histograms: the number of times each decision strategy wins; horizontal histograms: the number of times each model for the common-cause prior wins. Note that the heuristic, estimates-based strategy for unity judgments was overwhelmingly supported as it has been in previous studies[7].

S9 Model predictions and parametric bootstrapping

S9.1 Predicted probability distributions for localization responses

Given the best-fitting model and model parameters, for each participant, condition ($i = 1$: congruent; $i = 2$: incongruent), phase ($j = 1$: pre-learning; $j = 2$: post-learning) and each audiovisual stimulus pair AV_l ($l \in \{1, 2, ..., 16\}$), we computed the joint probability distribution, $P(m'_{AV_l,A}, m'_{AV_l,V}|s_{i,j,AV_l})$ by taking the outer product of the probability of visual measurements and the probability of auditory measurements, that is,

$$P(m'_{AV_l,A}, m'_{AV_l,V}|s_{i,j,AV_l}) \approx P(M_V = m'_{AV_l,V}|s_{i,j,AV_l}) \otimes P(M_A = m'_{AV_l,A}|s_{i,j,AV_l}), \tag{S25}$$

where M_A and M_V are random variables for auditory and visual measurements. Their ranges were selected to extend 5 standard deviations of the sensory noise away from the remapped stimulus location in perceptual space, that is, from $s'_{AV_l,A} - 5\sigma'_{AV,A}$ to $s'_{AV_l,A} + 5\sigma'_{AV,A}$ for auditory measurements, and from $s'_{AV_l,V} - 5\sigma'_{AV,V}$ to $s'_{AV_l,V} + 5\sigma'_{AV,V}$ for visual measurements. Both variables have 100 evenly spaced discrete values, resulting in a 100×100 grid (Fig. S6A).

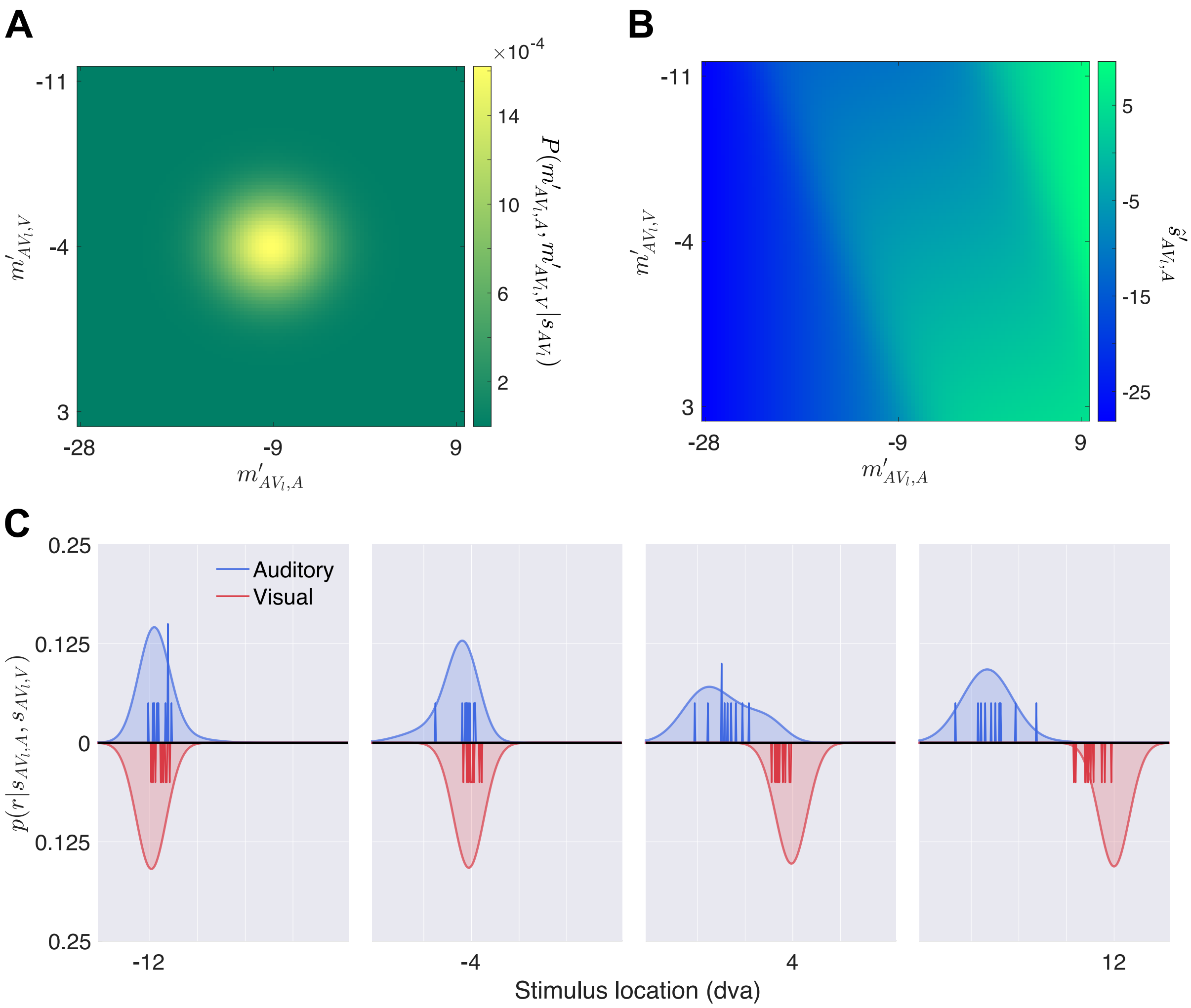

Figure S6. Model comparison. (A) Model predictions for localization responses. (A) The joint probability distribution of remapped auditory and visual measurements when participant YZ was presented with an auditory stimulus at $-12°$ and a visual stimulus at $-4°$ at the same time during the congruent pre-learning phase. (B) The final auditory location estimates, computed assuming model averaging, for each combination of remapped auditory and visual measurements. (C) Individual localization responses (solid lines) and predicted probability distributions (shaded areas) when participant YZ was presented with an auditory stimulus at $-12°$ and a visual stimulus at -12, -4, 4, and 12°, respectively for the congruent pre-learning phase. Red: visual; blue: auditory.

Then, for each combination of auditory and visual measurements, $M_A = m'_{AV_l,A}$ and $M_V = m'_{AV_l,V}$, we computed the final auditory location estimate based on the best-fitting decision strategy for each participant (Fig. S6B). We then computed the

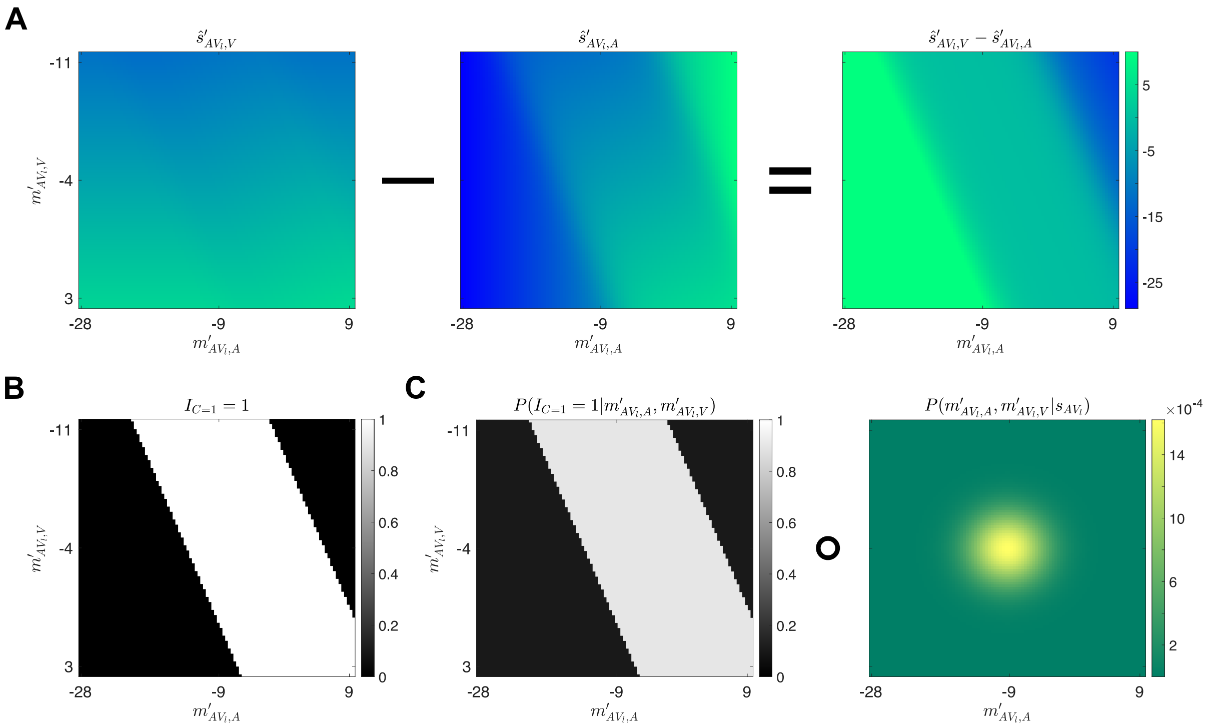

Figure S7. Model predictions for proportions of reporting 'common-cause'. (A) The difference (right panel) between the final visual (left panel) and auditory location estimates (middle panel) for each combination of remapped auditory and visual measurements. (B) The intended 'common-cause' response (white: $I_{C=1} = 1$; black: $I_{C=1} = 0$). (C) The actual proportion of 'common-cause' responses, which takes the lapse rate into account, is multiplied, element by element, by the joint probability distribution of remapped auditory and visual measurements.

leftmost and rightmost final location estimates and calculated the range, denoted as $\max(\hat{s}'_{AV_l,A}) - \min(\hat{s}'_{AV_l,A})$ and $\max(\hat{s}'_{AV_l,V}) - \min(\hat{s}'_{AV_l,V})$, respectively. We defined two new random variables, R_A and R_V, such that

$$P(R_A = r_A|s_{AV_l}) = \sum_{m=1}^{100}\sum_{n=1}^{100} \phi\left(r_A; \hat{s}'_{AV_l,A}(m,n), \sigma_r\right) P\left(m'_{AV_l,A}(m), m'_{AV_l,V}(n)|s_{AV_l}\right) \tag{S26}$$

and analogously,

$$P(R_V = r_V|s_{AV_l}) = \sum_{m=1}^{100}\sum_{n=1}^{100} \phi\left(r_V; \hat{s}'_{AV_l,V}(m,n), \sigma_r\right) P\left(m'_{AV_l,A}(m), m'_{AV_l,V}(n)|s_{AV_l}\right), \tag{S27}$$

where m and n index the discrete value for remapped auditory and visual measurements, respectively, and r_A ranges from $2\min(\hat{s}'_{AV_l,A}) - \max(\hat{s}'_{AV_l,A})$ to $2\max(\hat{s}'_{AV_l,A}) - \min(\hat{s}'_{AV_l,A})$ with an increment of 0.1. σ_r represents the variability in responses due to loss of precision in memory or the use of the response device (Eq. 12). In other words, the probability distribution of localization responses is the sum, across all possible combinations of auditory and visual measurements, of Gaussian distributions centered at the intended response location, with the variability being the motor/memory noise, weighted by the joint probability of the corresponding auditory and visual measurements (Fig. S6C).

Given the probability distribution of localization responses, we computed the model-predicted ventriloquism effects (VE) as follows:

$$VE_A = \left(\sum r_A P(R_A = r_A|s_{AV_l})\right) - \mu_{\hat{s}'_A} \tag{S28}$$

and

$$VE_A = \left(\sum r_V P(R_V = r_V|s_{AV_l})\right) - \mu_{\hat{s}'_V}, \tag{S29}$$

where $\mu_{\hat{s}'_A}$ and $\mu_{\hat{s}'_V}$ represent the mean perceived auditory stimulus locations when they were presented alone at location $s_{AV_l,A}$ and $s_{AV_l,V}$, respectively (Eq. 14). In other words, the model-predicted ventriloquism effect is the difference between the

expected value of the localization response for a given audiovisual stimulus pair s_{AV_l} and the mean perceived stimulus location when each element of s_{AV_l} was presented alone. The difference was coded as positive if the expected value of the localization response shifts in the direction of the other stimulus modality.

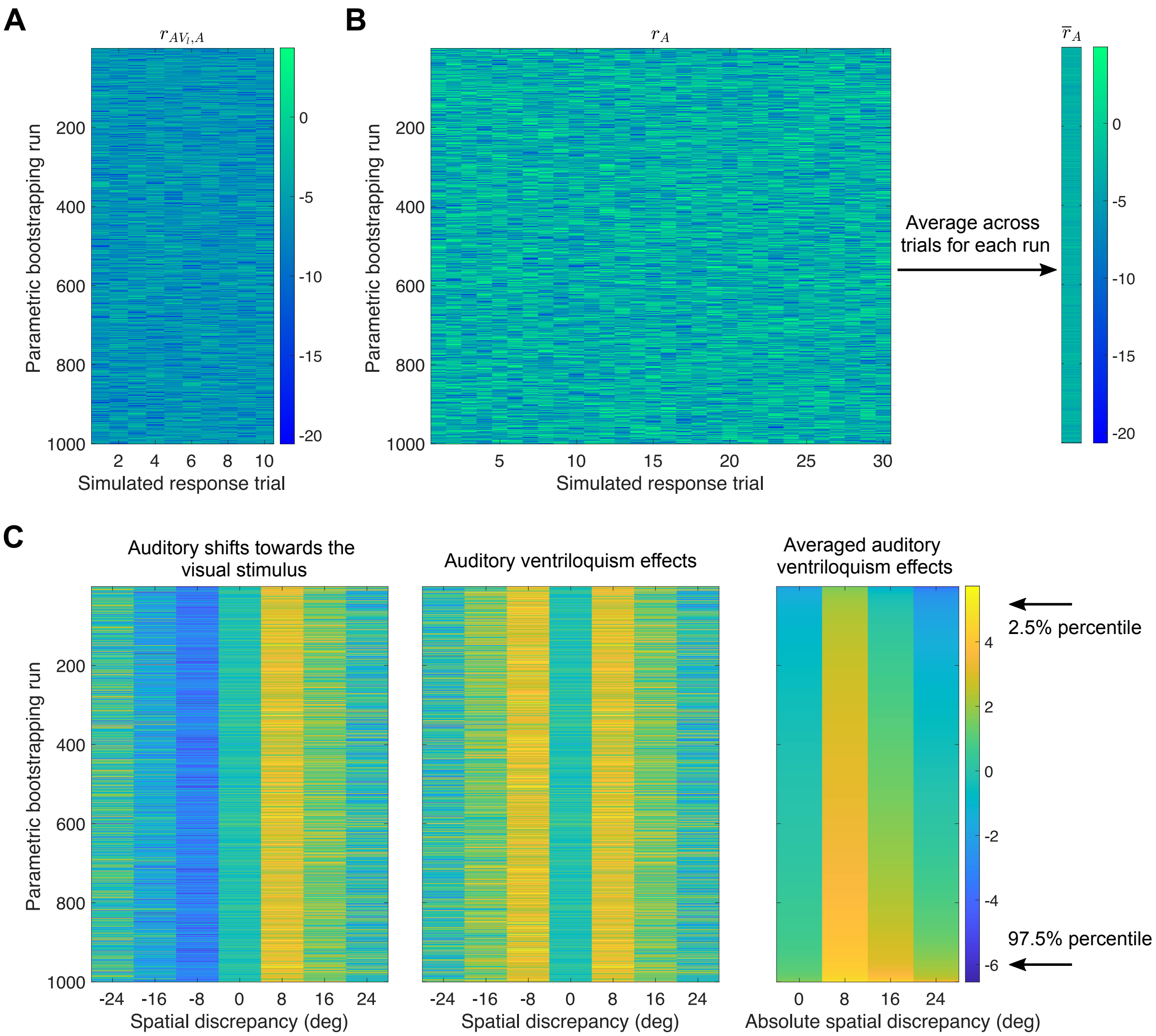

Figure S8. The procedure for computing 95% confidence intervals on auditory ventriloquism effects using parametric bootstrapping. (A) Simulated auditory localization responses given an audiovisual stimulus pair s_{AV_l}. (B) Simulated unimodal auditory localization responses when the auditory stimulus is presented alone at $s_{AV_l,A}$ (left panel), and the mean localization responses averaged across trials for each simulation run (right panel). (C) Auditory shifts towards the visual stimulus as a function of spatial discrepancy (left panel), and auditory ventriloquism effects, which are the same as auditory shifts but shifts towards the visual stimulus are coded as positive (middle panel), and (sorted) averaged auditory ventriloquism effects across trials with the same absolute spatial discrepancy (right panel). The lower and upper bounds of the 95% confidence intervals correspond to the 2.5th and 97.5th percentiles, respectively.

S9.2 Predicted proportion of reporting 'common cause'

Given each combination of auditory and visual measurements, the model-predicted probability of reporting 'common cause', $P(I_{C=1} = 1|m'_{AV_l,A}, m'_{AV_l,V})$, was computed using Eq. 11 if the best-fitting decision strategy is based on a comparison of the distance between auditory and visual final location estimates (Fig. S7A) and a participant-specific internal criterion. Specifically, if the distance is smaller than the internal criterion, then participants report 'common cause'; otherwise, they report 'separate causes' (Fig. S7B; Eq. S4 for the posterior-based decision strategy, and Eq. S5 for the measurements-based decision strategy).

The probability of reporting 'common cause' given an audiovisual stimulus pair s_{AV_l}, $P(I_{C=1} = 1|s_{AV_l})$ is computed as follows:

$$P(I_{C=1} = 1|s_{AV_l}) = \sum_{m=1}^{100}\sum_{n=1}^{100} P(I_{C=1} = 1|m'_{AV_l,A}, m'_{AV_l,V})P(m'_{AV_l,A}, m'_{AV_l,V}|s_{AV_l}). \tag{S30}$$

In other words, $P(I_{C=1} = 1|s_{AV_l})$ is the sum, across all possible combinations of auditory and visual measurements, of the probability of reporting 'common cause' by taking lapse rate into account, weighted by the joint probability of the corresponding auditory and visual measurements (Fig. S7C).

S9.3 Confidence intervals for model-predicted ventriloquism effects using parametric bootstrapping

To derive 95% confidence intervals on model-predicted ventriloquism effects, we used a method called parametric bootstrapping. Specifically, given the best-fitting model and model parameters, we first computed the probability distribution of auditory and visual localization responses for each audiovisual stimulus pair s_{AV_l}, phase, condition, and each participant (Eqs. 9-10, S13-S14). Then, we repeatedly drew ten localization responses from the probability distribution for each stimulus modality, $r_{AV_l,A}$ and $r_{AV_l,V}$ (Fig. S8A), and repeated this procedure 1000 times. Additionally, we repeatedly drew 30 unimodal localization responses for an auditory stimulus presented at $r_{AV_l,A}$ and for a visual stimulus presented at $r_{AV_l,V}$, respectively, based on closed-form formulas (Eq. 13), and computed the mean unimodal localization responses across trials for each of 1000 simulation runs (Fig. S8B). The number of trials for each simulation run was selected to match the actual number of trials used in our study.

We then calculated the auditory and visual shifts by subtracting the mean unimodal localization response (Fig. S8B) from the bimodal localization responses (Fig. S8A), and at the end took an average of the shifts across trials for each simulation run. We repeated this procedure for every audiovisual stimulus pair ($l \in \{1, 2, ..., 16\}$), and averaged the shifts of stimulus pairs that correspond to the same spatial discrepancy (e.g., four pairs have a spatial discrepancy of 0°: [$s_V = -12°$, $s_{A,1}$], [$s_V = -4°$, $s_{A,2}$], [$s_V = 4°$, $s_{A,3}$], and [$s_V = 12°$, $s_{A,4}$]; Fig. S8C, left panel). Ventriloquism effects are almost equivalent to the auditory and visual shifts, except that shifts towards the other stimulus modality were coded as positive (Fig. S8C, middle panel). Finally, we averaged the ventriloquism effects of stimulus pairs that correspond to the same absolute spatial discrepancy, sorted them independently for each absolute spatial discrepancy in ascending order, and computed the lower and upper bounds of the 95% confidence intervals, which correspond to the 2.5th and 97.5th percentiles (Fig. S8C, right panel).

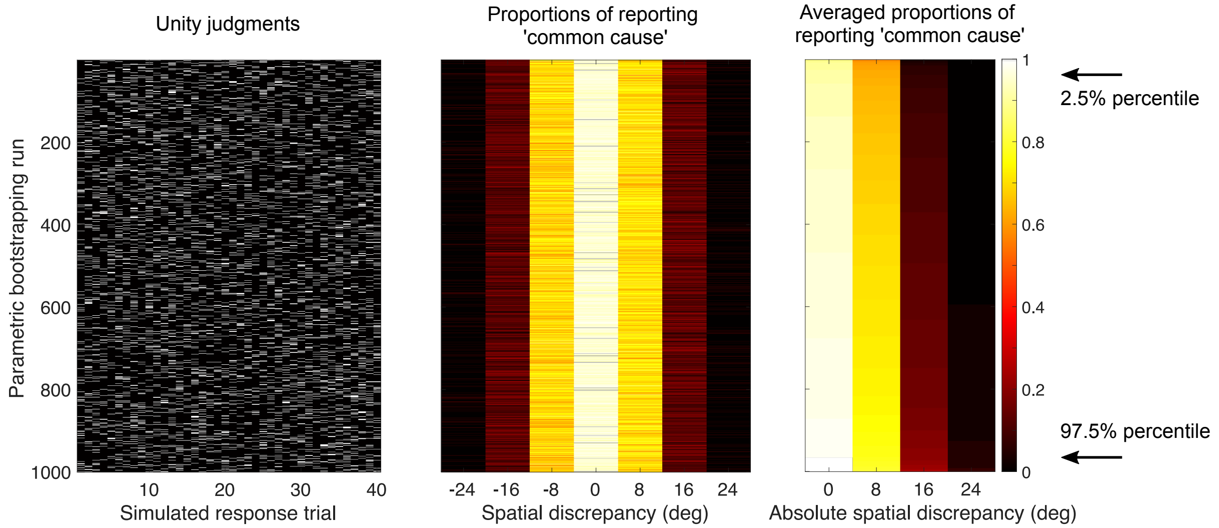

Figure S9. The procedure for computing 95% confidence intervals on proportions of reporting 'common cause'. Left panel: simulated binary responses (white: a 'common cause' response; black: a 'separate causes' response) for the audiovisual stimulus pair with a spatial discrepancy of −16°; middle panel: proportions of reporting 'common cause' as a function of spatial discrepancy; right panel: averaged proportions of reporting 'common cause' for audiovisual stimulus pairs that have the same absolute spatial discrepancy. The lower and upper bounds of the 95% confidence intervals correspond to the 2.5% and 97.5% percentiles.

S9.4 Confidence intervals for model-predicted proportions of reporting 'common cause' using parametric bootstrapping

To derive 95% confidence intervals on the proportion of reporting 'common cause', we again used parametric bootstrapping. Specifically, given the best-fitting model and model parameters, we first computed the proportion of reporting 'common cause'

for each audiovisual stimulus pair s_{AV_l}, phase, condition, and each participant (Eq. S30), and then averaged the proportions of reporting 'common cause' for audiovisual stimulus pairs that correspond to the same spatial discrepancy. Then, we drew N binary judgments (Fig. S9, left panel; $I_{C=1} = 1$: common cause; $I_{C=1} = 0$: separate causes) for each level of spatial discrepancy, where N represents the number of trials for each spatial discrepancy (N = 20, 40, 60, 80, 60, 40, 20 for spatial discrepancies of -24, -16, -8, 0, 8, 16, 24°). We repeated this procedure 1000 times. We computed the proportion of reporting 'common cause' across trials for each simulation run and each spatial discrepancy (Fig. S9, middle panel). Finally, we averaged the proportions of reporting 'common cause' of stimulus pairs that correspond to the same absolute spatial discrepancy, sorted them independently for each absolute spatial discrepancy in ascending order, and computed the lower and upper bounds of the 95% confidence intervals, which correspond to the 2.5th and 97.5th percentiles (Fig. S9, right panel).

S10 Simulated posterior probabilities of a common cause and changes of the common-cause prior given varying sensory measurement noise

S10.1 The posterior probability of a common cause

Table S10. Free parameters and their values for simulations shown in Fig. 6A-B.

Parameter	Meaning	Value	
		Fig. 6A	Fig. 6B
s_A	Auditory stimulus location	-2	-5
s_V	Visual stimulus location	2	-5 to 8 (100 steps)
a_A	The proportional bias in auditory spatial perception	1	
b_A	The constant bias in auditory spatial perception	0	
$p_{C=1}$	Common-cause prior	0.37	
σ'_A	The variability of auditory location measurements (in perceptual space)	0.1 to 5 (100 steps)	2.5
σ'_V	The variability of visual location measurements (in perceptual space)	0.01 to 2 (100 steps)	0.5
μ'_P	The center of a supra-modal prior over stimulus location	0	
σ'_P	The variability of a supra-modal prior over stimulus location	100	

S10.2 The updates of the common-cause prior

Table S11. Free parameters and their values for simulations shown in Fig. 6C-D.

Parameter	Meaning	Value	
		Congruent learning phase	Incongruent learning phase
s_A	Auditory stimulus location	-2.57, 0.64, 3.86, 7.07	
s_V	Visual stimulus location	-12, -4, 4, 12	s_{A_1}:[-27,3]<br>s_{A_2}:[-19,11]<br>s_{A_3}:[-11,19]<br>s_{A_4}:[-3,27]
a_A	The proportional bias in auditory spatial perception	2.49	
b_A	The constant bias in auditory spatial perception	-5.60	
$p_{C=1,pre}$	Common-cause prior	0.67	
$\alpha_{p_{C=1}}$	Updating rate of $p_{C=1}$	0.03	
$\sigma'_{AV,A}$	The variability of auditory location measurements (in perceptual space)	0.1 to 12.78 (21 steps)	1 to 18.26 (21 steps)
$\sigma'_{AV,V}$	The variability of visual location measurements (in perceptual space)	0.01 to 1.83 (21 steps)	0.1 to 3.93 (21 steps)
μ'_P	The center of a supra-modal prior over stimulus location	0	
σ'_P	The variability of a supra-modal prior over stimulus location	100	
$\tilde{a}_A$	Inaccurately estimated proportional bias in auditory spatial perception	Small: 1.87<br>Large: 1.25	
$\tilde{b}_A$	Inaccurately estimated constant bias in auditory spatial perception	Small: -8.60<br>Large: -11.60	

S10.3 The effects of the 'startup' common-cause prior on its updates

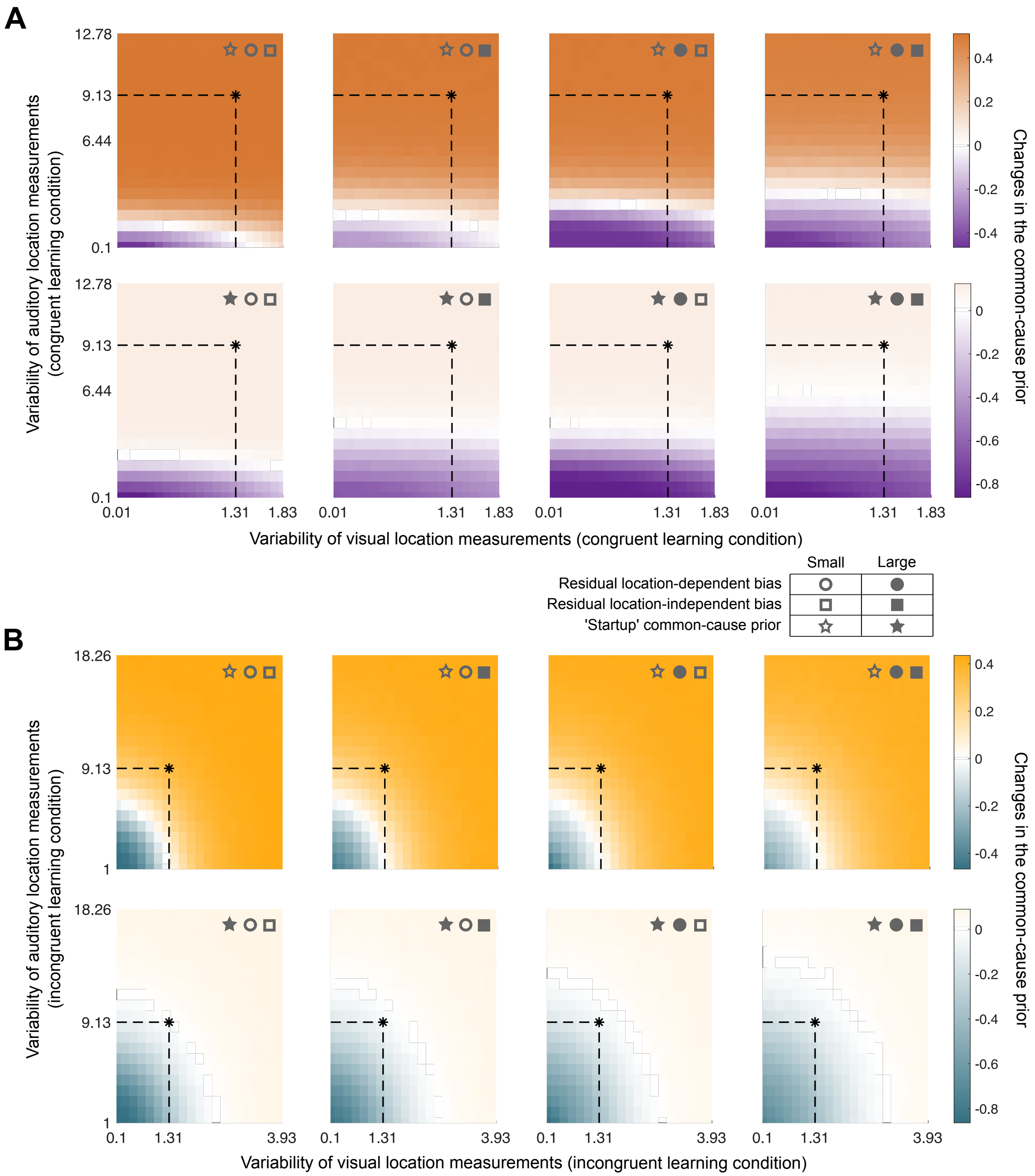

Figure S10. Simulations results for the congruent (A) and incongruent (B) learning conditions. Color key: accumulated changes in the common-cause prior, averaged across 100 simulations of repeated exposure to congruent/incongruent audiovisual stimulus pairs, given different variabilities of auditory (vertical axis) and visual (horizontal axis) location measurements, different residual perceptual biases, and different common-cause priors the simulated participant started with at the beginning the the learning phase (top row: $p_{C=1} = 0.27$; bottom row: $p_{C=1} = 0.87$).

References

1. Wichmann, F. A. & Hill, N. J. The psychometric function: I. fitting, sampling, and goodness of fit. *Percept Psychophys* **63**, 1293–313, DOI: 10.3758/bf03194544 (2001).
2. Lewald, J. Rapid adaptation to auditory-visual spatial disparity. *Learn. Mem* **9**, 268–78, DOI: 10.1101/lm.51402 (2002).
3. Lewald, J. & Ehrenstein, W. H. Auditory-visual spatial integration: a new psychophysical approach using laser pointing to acoustic targets. *J Acoust Soc Am* **104**, 1586–97, DOI: 10.1121/1.424371 (1998).
4. Odegaard, B., Wozny, D. R. & Shams, L. Biases in visual, auditory, and audiovisual perception of space. *PLoS Comput. Biol* **11**, e1004649, DOI: 10.1371/journal.pcbi.1004649 (2015).
5. Parise, C. V., Spence, C. & Ernst, M. O. When correlation implies causation in multisensory integration. *Curr Biol* **22**, 46–9, DOI: 10.1016/j.cub.2011.11.039 (2012).
6. Wozny, D. R., Beierholm, U. R. & Shams, L. Probability matching as a computational strategy used in perception. *PLoS Comput. Biol* **6**, DOI: 10.1371/journal.pcbi.1000871 (2010).
7. Badde, S., Navarro, K. T. & Landy, M. S. Modality-specific attention attenuates visual-tactile integration and recalibration effects by reducing prior expectations of a common source for vision and touch. *Cognition* **197**, 104170, DOI: 10.1016/j.cognition.2019.104170 (2020).
